# The Zn_2_Cys_6_-type transcription factor LeuB cross-links regulation of leucine biosynthesis and iron acquisition in *Aspergillus fumigatus*

**DOI:** 10.1371/journal.pgen.1007762

**Published:** 2018-10-26

**Authors:** Nanbiao Long, Thomas Orasch, Shizhu Zhang, Lu Gao, Xiaoling Xu, Peter Hortschansky, Jing Ye, Fenli Zhang, Kai Xu, Fabio Gsaller, Maria Straßburger, Ulrike Binder, Thorsten Heinekamp, Axel A. Brakhage, Hubertus Haas, Ling Lu

**Affiliations:** 1 Jiangsu Key Laboratory for Microbes and Functional Genomics, College of Life Sciences, Nanjing Normal University, Nanjing, China; 2 Department of Laboratory Medicine, Shaoyang University, Shaoyang, China; 3 Division of Molecular Biology/Biocenter, Medical University of Innsbruck, Innrain, Innsbruck, Austria; 4 Department of Molecular and Applied Microbiology, Leibniz Institute for Natural Product Research and Infection Biology (HKI), and Friedrich Schiller University Jena, Jena, Germany; 5 Transfer Group Anti-infectives, Leibniz Institute for Natural Product Research and Infection Biology (HKI), Jena, Germany; 6 Division of Hygiene & Medical Microbiology, Medical University of Innsbruck, Innsbruck, Austria; Oregon State University, UNITED STATES

## Abstract

Both branched-chain amino acids (BCAA) and iron are essential nutrients for eukaryotic cells. Previously, the Zn_2_Cys_6_-type transcription factor Leu3/LeuB was shown to play a crucial role in regulation of BCAA biosynthesis and nitrogen metabolism in *Saccharomyces cerevisiae* and *Aspergillus nidulans*. In this study, we found that the *A*. *fumigatus* homolog LeuB is involved in regulation of not only BCAA biosynthesis and nitrogen metabolism but also iron acquisition including siderophore metabolism. Lack of LeuB caused a growth defect, which was cured by supplementation with leucine or iron. Moreover, simultaneous inactivation of LeuB and HapX, a bZIP transcription factor required for adaptation to iron starvation, significantly aggravated the growth defect caused by inactivation of one of these regulators during iron starvation. In agreement with a direct role in regulation of both BCAA and iron metabolism, LeuB was found to bind to phylogenetically conserved motifs in promoters of genes involved in BCAA biosynthesis, nitrogen metabolism, and iron acquisition *in vitro* and *in vivo*, and was required for full activation of their expression. Lack of LeuB also caused activation of protease activity and autophagy *via* leucine depletion. Moreover, LeuB inactivation resulted in virulence attenuation of *A*. *fumigatus* in *Galleria mellonella*. Taken together, this study identified a previously uncharacterized direct cross-regulation of BCCA biosynthesis, nitrogen metabolism and iron homeostasis as well as proteolysis.

## Author summary

Adaptation to the host niche is an essential attribute of pathogens. Here we found that the Zn_2_Cys_6_-type transcription factor LeuB cross-regulates branched-chain amino acid biosynthesis, nitrogen metabolism, iron acquisition *via* siderophores, and proteasome activity in the mold *Aspergillus fumigatus*. Lack of this regulatory circuit impaired virulence in an insect infection model. Mammals do neither express Zn_2_Cys_6_-type transcription factors nor have the capacity to produce branched-chain amino acids or siderophores. Consequently, this regulatory circuit is a paradigm for fungal pathogen-specific adaptation to the host niche.

## Introduction

*Aspergillus fumigatus* is the most important airborne fungal pathogen, causing allergic and invasive diseases, termed aspergillosis, the latter particularly in immunocompromised patients [1,2]. A critical virulence attribute of most pathogens, including *A*. *fumigatus*, is efficient iron acquisition [3–6]. On one hand, iron is an essential cofactor required for a large number of biological processes including respiration and biosynthesis of deoxyribonucleic acid, amino acids and lipids. On the other hand, mammalian hosts represent an iron-limited niche as the iron is tightly sequestered by proteins and, moreover, the innate immune system responds with iron-withholding strategies to infections [7]. Therefore, to overcome the low bioavailability of iron during invasion, pathogens have evolved various systems to struggle for iron from host. *A*. *fumigatus* possesses two major high-affinity iron uptake systems, reductive iron assimilation (RIA) and siderophore-mediated iron acquisition (SIA), whereby the latter has been shown to be essential for virulence [8,9]. Siderophores are low-molecular mass, ferric iron specific chelators, the production and secretion of which is induced by iron starvation. The major secreted siderophore of *A*. *fumigatus* is triacetylfusarinine C (TAFC). Upon binding of environmental iron, the TAFC-iron chelate is taken up by a specific transporter, termed MirB [10,11]. Reverse genetics identified several SIA components, which proved to be crucial for virulence of *A*. *fumigatus* in animal models of aspergillosis [12–15]. As iron is not only essential but also toxic in excess, iron uptake, consumption and detoxification have to be tightly controlled. To maintain iron homeostasis, *A*. *fumigatus* has evolved two major transcription factors, SreA and HapX. The GATA-type transcription factor SreA represses RIA and SIA during sufficient iron supply [16]. In contrast, the bZIP-type transcription factor HapX represses iron-dependent pathways to spare iron and activates RIA and SIA to promote iron uptake during iron starvation [17], while it activates iron-dependent pathways and particular iron detoxification *via* transport into the vacuole during iron excess [18].

In *A*. *fumigatus*, one of the iron-dependent pathways comprising genes repressed during iron starvation and induced by iron excess *via* HapX is branched-chain amino acid (BCAA) biosynthesis [17]. Mammals do not have the capacity to produce BCAA and thus valine, leucine or isoleucine must be supplied in the diet. In contrast, fungi are BCAA autonomous. As depicted in S1 Fig (using the nomenclature for *S*. *cerevisiae*), BCAA biosynthesis consists of a common pathway that leads from pyruvate and threonine, respectively, to valine and isoleucine. Leucine biosynthesis starts with an intermediate of valine biosynthesis, 2-ketoisovalerate, which is converted to α-isopropylmalate (αIPM) by αIPM synthase (*S*. *cerevisae* contains two paralogs Leu4/9). αIPM is then further processed by the αIPM isomerase (Leu1), the β-isopropylmalate (βIPM) dehydrogenase (Leu2), and the branched-chain amino acid aminotransferase (Bat2). Leucine biosynthesis is feedback-regulated due to inhibition of Leu4/9 enzyme activity by leucine. Moreover, in *S*. *cerevisiae* the intermediate αIPM has been shown to posttranslationally activate the Zn_2_Cys_6_-type transcription factor Leu3, which activates several steps in BCAA biosynthesis (Ilv2 and Ilv5, which are involved in general BCAA biosynthesis, and the leucine biosynthetic enzymes Leu4, Leu1, Leu2 and Bat1) as well as the NADP-dependent glutamate dehydrogenase Gdh1, a crucial enzyme in nitrogen metabolism [19]. Likewise, the Leu3 homolog LeuB transcriptionally activates genes involved in leucine biosynthesis as well as the glutamate dehydrogenase-encoding gene *gdhA* in *Aspergillus nidulans* [19–21]. The BCAA biosynthetic pathway comprises two enzymes, whose activity depends on iron-sulfur clusters: mitochondrial dihydroxyacid dehydratase (termed Ilv3 in *S*. *cerevisiae*), which is required for biosynthesis of all three BCAAs, as well as cytosolic 3-isopropylmalate dehydratase (termed Leu1 in *S*. *cerevisiae*), which is required exclusively for biosynthesis of leucine [19]. In *A*. *fumigatus*, these two enzymes were found to be transcriptionally downregulated during iron starvation together with numerous other “iron-dependent genes” in order to spare iron [17].

In the current study, we identified in *A*. *fumigatus* a cross-regulatory link of BCAA biosynthesis, nitrogen metabolism and iron homeostasis *via* the transcription factor LeuB (AFUB_020530).

## Results

### LeuB binds *in vitro* to phylogenetically conserved motifs in the promoters of *hapX* and BCAA biosynthetic genes

**Fig 1:**
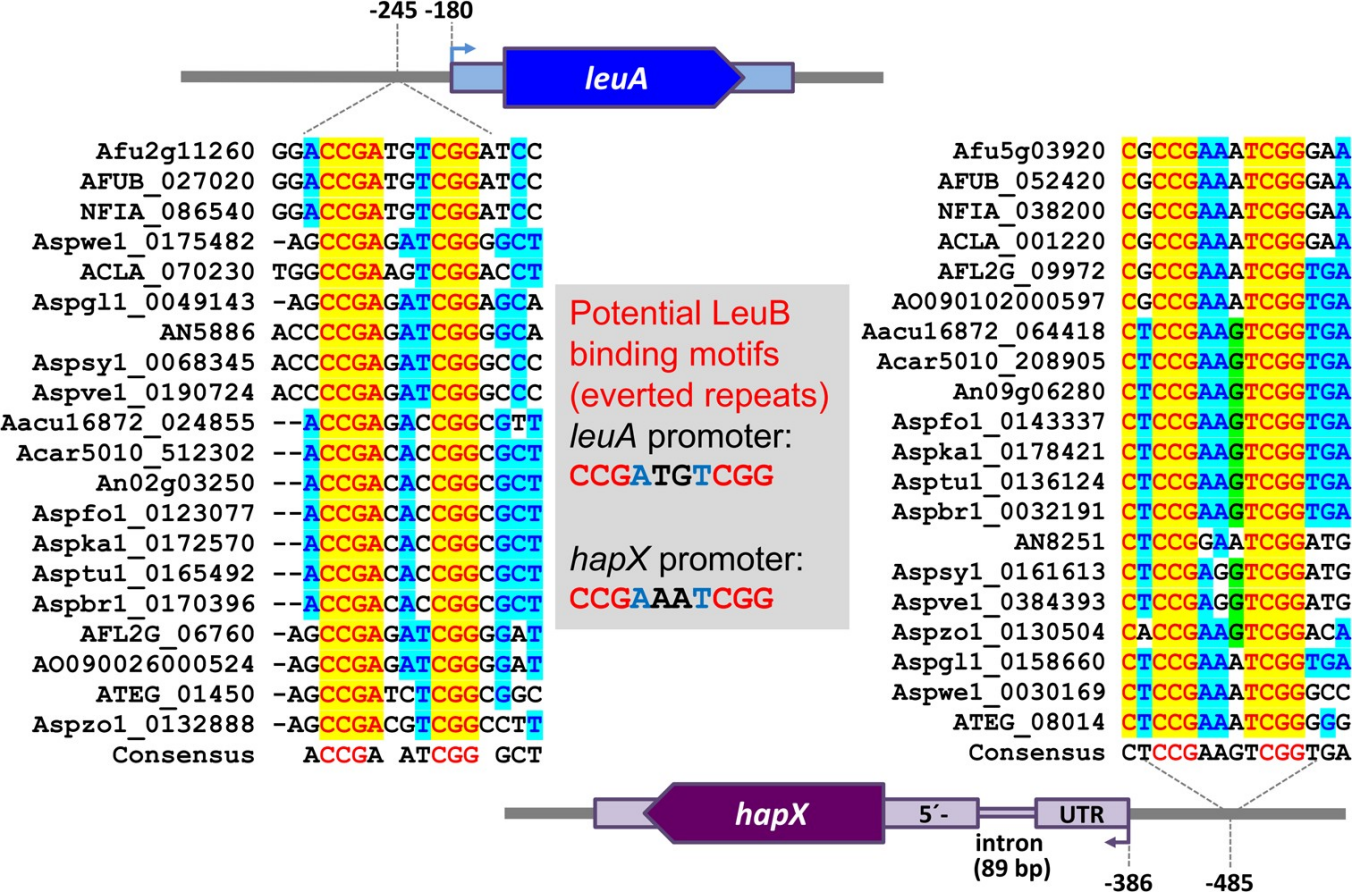
The promoters of the BCAA biosynthetic *leuA* and the iron-regulatory *hapX* genes contain an evolutionary conserved CCGN_4_CGG motif. MEME analysis was conducted with the following species including strain designation: *A*. *nidulans* FGSC A4, *A*. *fumigatus* Af293, *A*. *fumigatus* A1163, *A*. *oryzae* RIB40/ATCC 42149, *A*. *flavus* NRRL 3357, *A*. *niger* CBS 513.88, *A*. *niger* ATCC 1015, *Neosartorya fischeri* NRRL 181, *A*. *clavatus* NRRL 1, *A*. *terreus* NIH2624, *A*. *acidus* CBS 106.47, *A*. *aculeatus* ATCC16872, *A*. *brasiliensis* CBS 101740, *A*. *carbonarius* ITEM 5010, *A*. *sydowii*, *A*. *versicolor*, *A*. *glaucus* CBS 516.65, *A*. *tubingensis* CBS 134.48, *A*. *wentii* DTO 134E9, *A*. *zonatus*, *A*. *kawachii* IFO 4308. Sequences were downloaded from AspGD (http://www.aspergillusgenome.org).

HapX is a key transcription factor coordinating the response to iron starvation. To identify potential regulators controlling expression of this transcription factor, we compared promoter regions of HapX-encoding genes. MEME analysis [22] of 1-kb 5’-upstream regions of *hapX* homologs from 20 *Aspergillus* species identified a highly conserved CCGN_4_CGG motif localized about 500 nt upstream of the *hapX* translation start point in *A*. *fumigatus* (Fig 1). This motif resembles the typical binding consensus sequence for Leu3/LeuB transcription factors [23]. In agreement, MEME analysis identified phylogenetically conserved CCGN_4_CGG motifs in promoters of several BCAA biosynthetic genes, i.e. α-isopropylmalate isomerase-encoding *leuA* (Fig 1), acetohydroxy acid reductoisomerase-encoding *ilv5*, α-isopropylmalate synthase-encoding *leuC* (homolog of *S*. *cerevisiae leu4/9*), and β-isopropylmalate dehydrogenase-encoding *leu2A* (homolog of *S*. *cerevisiae leu2*) as well as glutamate dehydrogenase-encoding *gdhA* (S1 Table). These findings suggested a role of LeuB in regulation of iron homeostasis in addition to BCAA biosynthesis in *A*. *fumigatus*.

**Fig 2:**
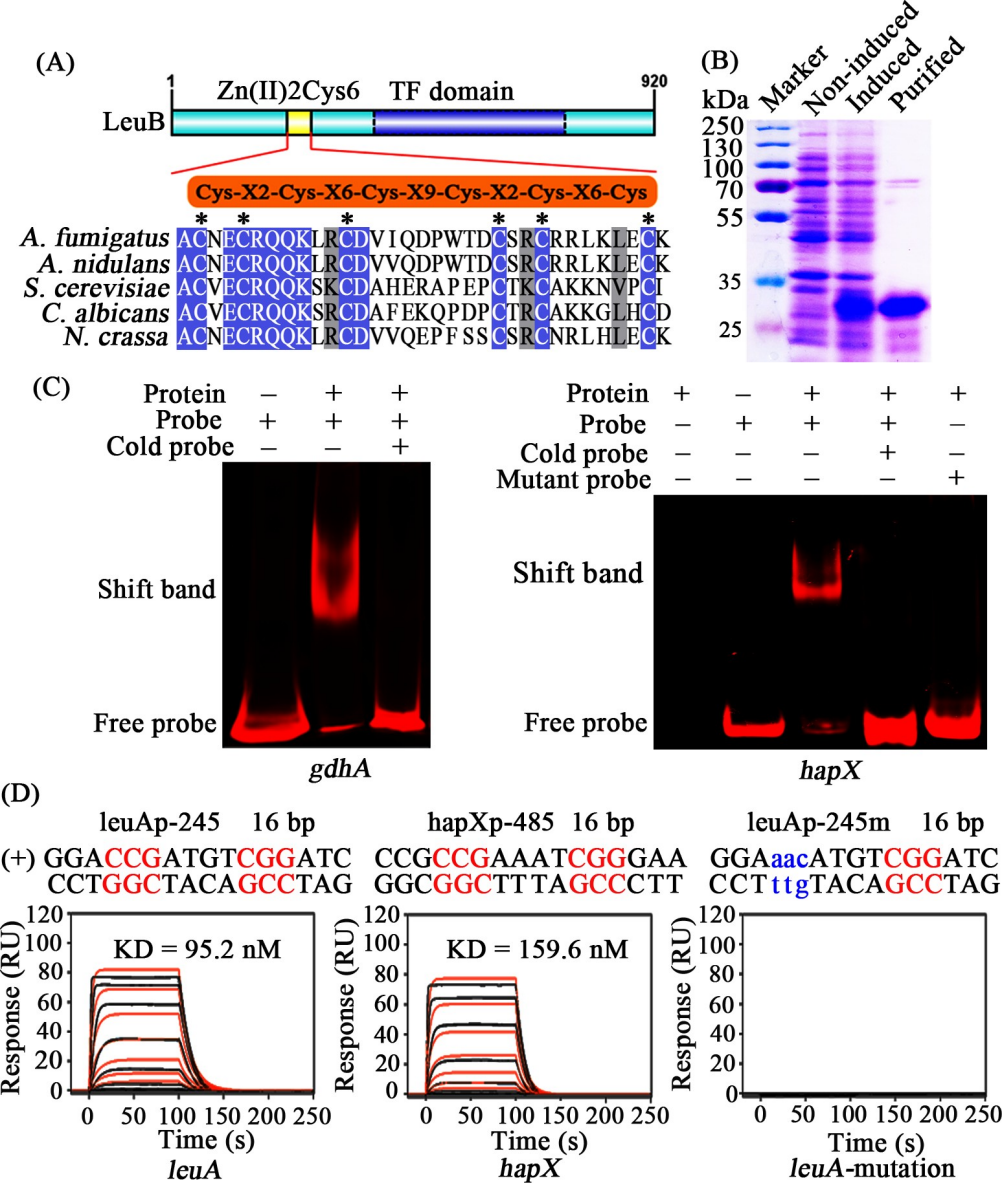
LeuB is able to bind to the CCGN_4_CGG motifs in the promoters of *gdhA*, *hapX* and *leuA*. (A) Zn_2_Cys_6_ domain alignment of the indicated species. (B) SDS-PAGE analysis of the recombinant LeuB DNA-binding domain produced in *E*. *coli*. (C) EMSA of LeuB binding to Cy5-labeled promoter fragments of *gdhA* and *hapX*. (D) Real-time *in vitro* SPR interaction analysis of the LeuB DNA-binding domain with DNA containing the predicted natural or mutant binding sites from *leuA* and *hapX* promoters. Sequences of DNA duplexes used for SPR analysis are shown on top of the sensorgrams. Numbers represent the CGG everted repeat motif positions relative to the start of the open reading frame. CGG half-sites are highlighted in red. Substituted nucleotides are shown in blue and lower case. LeuB binding responses injected in duplicate (black lines) are overlaid with the best fit derived from a 1:1 interaction model including a mass transport term (red lines). Dissociation constants (K_D_) are plotted inside the sensorgrams.

BLAST analysis identified an *A*. *fumigatus* homolog (AFUB_020530/ AFUA_2G03460) to *A*. *nidulans* LeuB (termed AnLeuB here). To investigate whether *A*. *fumigatus* LeuB (termed LeuB here) can directly interact with the predicted CCGN_4_CGG motifs, we expressed the predicted DNA binding domain of LeuB in *E*. *coli* and analyzed protein-DNA interaction (Fig 2A and 2B). As shown in Fig 2C, LeuB clearly interacted with promoter fragments of *hapX* and *gdhA* genes in electrophoretic mobility shift assays (EMSA). The *gdhA* gene encodes NADP-dependent glutamate dehydrogenase, a key enzyme in nitrogen metabolism, which has previously been shown to be regulated by Leu3/LeuB in *S*. *cerevisiae* and *A*. *nidulans* [19–21]. Excess of unlabeled DNA and mutating the CCGN_4_CGG motif to CTGN_4_CAG in the *hapX* fragment blocked the interaction of LeuB with the promoter fragments, underlining the specificity of the protein/DNA interaction.

Surface plasmon resonance (SPR) analyses, shown in Fig 2D, confirmed that the DNA-binding domain of LeuB binds with high affinity to the phylogenetically conserved CCGN_4_CGG motifs in the promoters of *hapX* (K_D_ = 159.6 nM) and *leuA* (K_D_ = 95.2 nM) and that mutation of one of the CCG sequences abolishes binding, which again underlines the sequence specificity.

### Nucleus-localized LeuB is involved in both BCAA biosynthesis and adaptation to iron starvation

**Fig 3:**
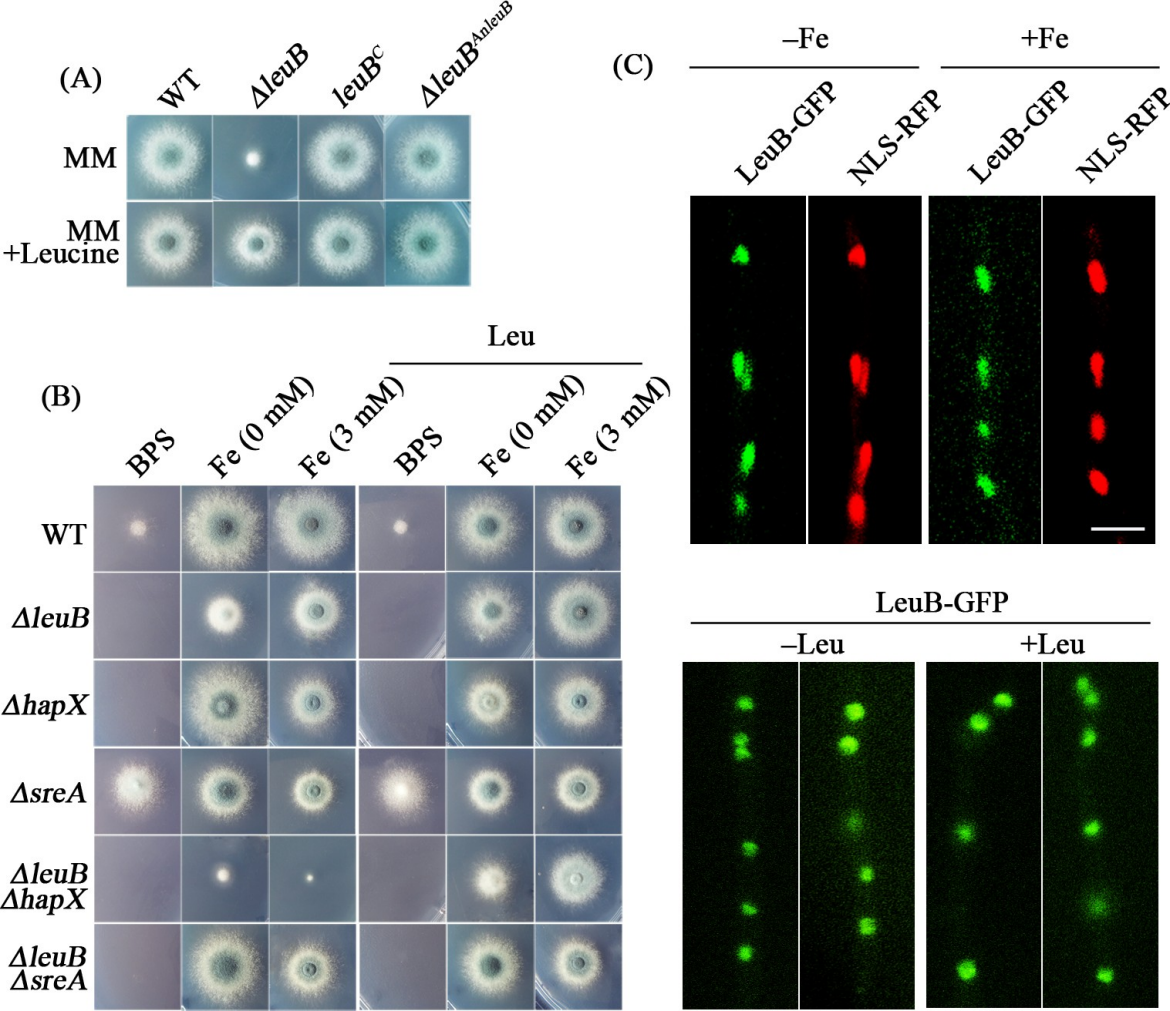
**Lack of LeuB results in iron- and leucine-dependent growth defects (A) and LeuB is localized in the nucleus independent of availability of iron and leucine (B).** (A) Phenotypes of the WT, *ΔleuB*, *leuB*^C^, *ΔleuB*^AnleuB^ strains on minimal medium with or without leucine supplementation (2 mM). (B) Phenotype of WT, *ΔleuB*, *ΔhapX*, *ΔsreA*, *ΔleuBΔhapX* and *ΔleuBΔsreA* strains on minimal medium in the presence of the iron chelator BPS (150 μM) or in the presence of the indicated concentrations of iron with or without supplementation with leucine (2 mM). (C) Epi-fluorescence microscopy demonstrating that LeuB tagged C-terminally with GFP was localized in the nucleus with addition of Fe (+ 50 μM), leucine (+ 2 mM) or without either of them. RFP-NLS indicates that the red fluorescence protein fused with a nuclear localization signal was used to visualize the nucleus. Scale bar = 10 μm.

To further analyze the function of LeuB in *A*. *fumigatus*, we generated a *leuB* gene deletion mutant, *ΔleuB*, by replacing the *leuB*-coding region with the *Neurospora crassa pyr4* gene. Compared to the parental wild-type (WT) strain, *ΔleuB* displayed dramatically reduced growth on minimal medium agar plates (Fig 3A). This growth defect was cured by re-integration of the *leuB* gene (strain *leuB*^*C*^) (Fig 3A), together with diagnostic PCR and Southern blot analyses which underline the accuracy of the genetic manipulation (S2 Fig). Moreover, leucine supplementation largely rescued the *ΔleuB* growth defect (Fig 3A), as previously seen in *S*. *cerevisiae* and *A*. *nidulans* mutants lacking orthologs of LeuB [19–21]. According to the hypothesis that LeuB might play a role in iron homeostasis, we analyzed the growth of *ΔleuB* under conditions of different iron availability (Fig 3B). Remarkably, the presence of the ferrous iron-specific chelator bathophenanthroline disulfonic acid (BPS), which inhibits RIA and creates an iron-poor environment [12], completely blocked growth of *ΔleuB*, while iron supplementation significantly improved the growth of *ΔleuB*. In agreement with LeuB being important for adaptation to iron starvation, the biomass of *ΔleuB* reached only 23.5% of that of the WT during iron starvation but increased to 70.8% during iron sufficiency in submersed culture conditions (S3A Fig). In contrast, other metals such as magnesium, calcium or manganese were unable to rescue the growth defects of *ΔleuB* (S3B Fig).

To investigate, whether LeuB is also involved in iron homeostasis in *A*. *nidulans*, we generated a *leuB* deletion mutant in *A*. *nidulans*, *ΔAnleuB*. Similar to *A*. *fumigatus*, the growth of *ΔAnleuB* was significantly stimulated by iron supplementation (S3C Fig). Moreover, the growth defect of the *A*. *fumigatus ΔleuB* mutant was rescued by genetic complementation with the *A*. *nidulans leuB* gene (strain *ΔleuB*^*AnleuB*^) (Fig 3A). These data document that the function of LeuB in iron homeostasis is conserved in *A*. *nidulans*.

The major transcription factors ensuring maintenance of iron homeostasis are SreA and HapX. SreA represses iron acquisition under sufficient iron supply and is consequently important for adaptation to iron excess [16]. HapX induces iron acquisition and represses iron consumption during iron starvation, while it induces iron detoxification during iron excess [17,18]. Consequently, HapX is important for adaptation to both iron starvation and iron excess. To further explore the role of LeuB in iron homeostasis, we generated mutants lacking either *leuB* and *hapX* (strain *ΔleuBΔhapX*) or *leuB* and *sreA* (strain *ΔleuBΔsreA*). Lack of both HapX and LeuB exacerbated the growth defect seen in mutants lacking only one of these two transcription factors, *i*.*e*. growth could not be cured by supplementation with high amounts of iron but only by leucine supplementation (Figs 3B and S3A). In comparison, *ΔleuBΔsreA* displayed improved growth under low iron conditions (BPS, without iron supplementation) compared to *ΔleuB* (Figs 3B and S3A). The suppression of the low-iron growth defect of *ΔleuB* by lack of SreA can be explained by the fact that lack of SreA derepresses iron acquisition and thereby increases iron uptake resembling supplementation with iron.

Taken together, these data collectively suggest that LeuB links iron homeostasis maintenance and leucine biosynthesis. Since LeuB is a putative transcription factor, we next analyzed its cellular localization *via* C-terminal tagging of LeuB with green fluorescent protein (LeuB-GFP protein) expressed under control of the native *leuB* promoter. Epi-fluorescence microscopy studies revealed predominant nuclear localization of LeuB-GFP during both iron starvation and sufficiency as well as with and without leucine supplementation (Fig 3C), suggesting posttranslational activation of LeuB independent of its intracellular localization.

### During iron starvation, lack of LeuB leads to increased cellular protease activity and increased autophagy due to leucine shortage

**Fig 4:**
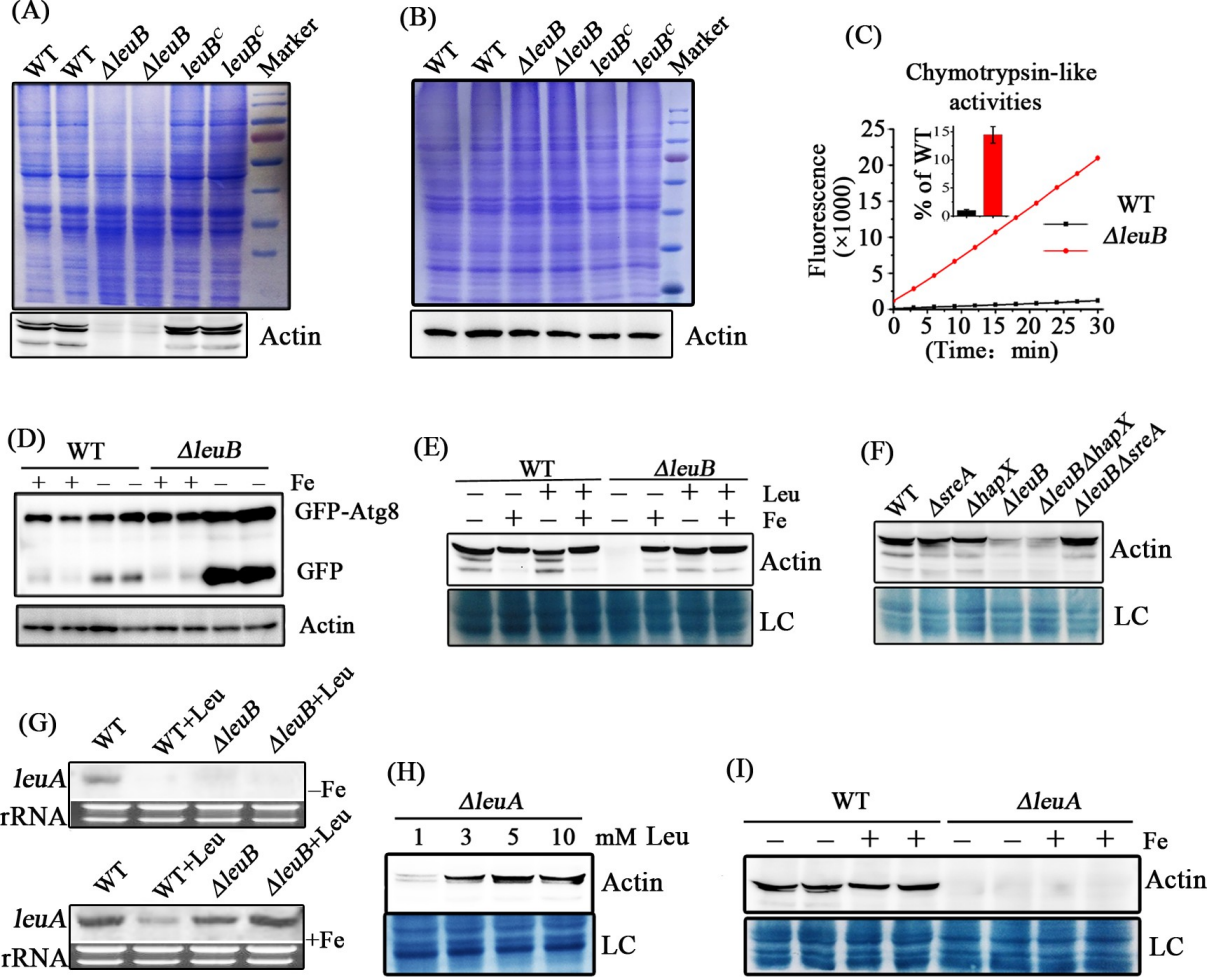
Lack of LeuB results in increased proteolytic activity during iron starvation caused by leucine shortage. *A*. *fumigatus* protein extracts were generated after growth for 24 hours during iron starvation (-Fe), unless iron supplementation (+Fe) is denoted, using a non-denaturing lysis buffer with exception of B and D, where a denaturing lysis buffer was used. In A, B, D and I, biological duplicates are shown. Leucine supplementation is denoted with Leu. (A) Compared to WT and *leuB*^C^, *ΔleuB* displays reduced abundance of high-molecular mass proteins and reduced amounts of actin during iron starvation as shown by SDS-PAGE analysis followed by Coomassie blue protein staining (above) and Western blot analysis (below) using an antibody recognizing the fungal housekeeping protein actin, respectively. (B) Using a denaturing lysis buffer, the increased protease activity observed in A is not seen indicating that the increased proteolysis identified above proceeds during protein extraction and not *in vivo*. (C) Protease activity assay demonstrating that lack of LeuB significantly increases chymotrypsin-like protease activity during iron starvation. (D) Western blot analysis of GFP-Atg8, employing a GFP-specific antibody, revealed dramatically increased cleavage of GFP from GFP-Atg8 in *ΔleuB* compared to WT during iron starvation, which is indicative for increased autophagy. Protein extraction was performed with a denaturing buffer. Actin showed similar abundance independent of iron availability in WT and *ΔleuB*, confirming that the increased proteolysis identified above proceeds during protein extraction and not *in vivo*. Moreover, these data demonstrate that cleavage of GFP-Atg8 takes place *in vivo* and not during protein extraction. (E) Western blot analysis of actin demonstrating that increased proteolytic activity in *ΔleuB* is cured by supplementation with leucine (2 mM) or iron (50 μM). (F) Lack of SreA but not HapX cures the increased proteolytic activity in *ΔleuB* protein extracts. (G) Northern blot analyses demonstrating that lack of LeuB significantly decreases *leuA* transcript levels during iron starvation but not iron sufficiency. (H) Western blot analysis of actin demonstrating that lack of LeuA results in increased proteolytic activity during iron starvation with limited (1mM) but not high leucine supplementation (5 and 10 mM). (I) Western blot analysis of actin demonstrating that the proteolytic activity caused by lack of LeuA with 1 mM leucine supplementation is not rescued by iron supplementation (50 μM).

Analysis of whole-cell protein extracts, using a non-denaturing extraction buffer, *via* sodium dodecylsulfate polyacrylamide gel electrophoresis (SDS-PAGE) followed by Coomassie blue-protein staining, revealed increased proteolysis in *ΔleuB* compared to WT and *leuB*^*c*^ strains, *i*.*e*., large-size proteins displayed significantly decreased abundance in mycelia cultured under iron starvation (Fig 4A). In agreement, Western blot analysis revealed significantly decreased abundance of the house-keeping protein actin in *ΔleuB* compared to the control strains (Fig 4A). Increased proteolysis in the *ΔleuB* whole-cell lysates from iron starved mycelia was further confirmed by a biochemical protease activity assay (Fig 4C).

In contrast, analysis of whole-cell lysates using a denaturing lysis buffer (alkaline lysis: 0.2 M NaOH, 0.2% β-mercaptoethanol) did not result in significant differences between *ΔleuB*, WT and *leuB*^*c*^ strains in SDS-PAGE analysis and actin did not show significant differences in abundance (Fig 4B). Taken together, these data suggest that the increased proteolysis in *ΔleuB* did not occur *in vivo* but during protein extraction due to the increased protease content of the cell extract. Increased protease activity is often coupled with increased autophagy. To analyze whether lack of LeuB induces autophagy, we tagged the autophagy marker protein Atg8 N-terminally with GFP in *ΔleuB* and WT genetic backgrounds. As shown in Fig 4D, Western blot analysis indicated that the WT displayed slightly increased cleavage of GFP from GFP-ATG8 during iron starvation compared to iron sufficiency, which indicates slightly increased autophagy [24,25]. In the *ΔleuB* genetic background, GFP-cleavage was WT-like during iron sufficiency but significantly increased during iron starvation compared to the WT genetic background. Consistently, epi-fluorescence microscopy studies displayed increased accumulation of GFP-ATG8 in vacuoles in *ΔleuB* compared to WT under iron starvation (S4 Fig). These data collectively demonstrate that lack of LeuB increases not only protease activity but also autophagy.

As supplementation with iron or leucine rescued the growth phenotype of *ΔleuB*, we investigated if these supplementations affect the protease activity in *ΔleuB* extracts. As shown in Fig 4E, supplementation with either iron (50 μM FeCl_3_), leucine (2 mM), or both decreased the protease activity, as illustrated by Western blot analysis of actin. In contrast to *ΔleuB*, *ΔsreA* and *ΔhapX* displayed WT-like protein stability of actin in cell extracts during iron starvation (Fig 4F), demonstrating that these iron regulators are not involved in control of the protease activity found in *ΔleuB* extracts. The *ΔleuBΔhapX* mutant strain showed *ΔleuB*-like proteolysis, while lack of SreA suppressed protease activity caused by lack of LeuB (*ΔleuBΔsreA*) (Fig 4F). The latter can be explained by the fact that lack of SreA derepresses iron acquisition and thereby increases iron uptake, which resembles the effect of iron supplementation [16]. In agreement, lack of SreA also cured the growth defect of *ΔleuB* (Fig 3B). Hence, the growth defects largely match with the increased protease activity in cell extracts.

As shown above, supplementation with iron or leucine rescues the *ΔleuB* growth phenotype and protease activity (Fig 3B and 4E). As iron starvation did not result in increased protease activity in the WT (Fig 4E), we hypothesized that the increased protease activity of *ΔleuB* extracts is caused by leucine shortage, particularly, as LeuB orthologs were reported to be involved in control of BCAA biosynthesis [19]. To test this hypothesis, we first analyzed expression of *leuA*, an essential gene for leucine biosynthesis, which contains a CCGN_4_CGG promoter motif that is bound *in vitro* with high affinity by LeuB. Transcript levels of *leuA* were significantly higher in WT during iron sufficiency compared to iron starvation (Fig 4G). This is in line with previous studies showing that expression of *leuA* is repressed during iron starvation by HapX [18]. Lack of LeuB caused a significant decrease of the *leuA* transcript levels during iron starvation but did not significantly affect *leuA* transcript levels during iron sufficiency (Fig 4G), suggesting that LeuB is a transcriptional activator of *leuA* expression particularly during iron starvation. Similarly, the *leuA* ortholog *leu1* has previously been shown to be regulated by the LeuB ortholog Leu3 in *S*. *cerevisiae* [26]. The decreased *leuA* transcript levels indicate a reduced leucine content in *ΔleuB*. To further analyze if decreased leucine content increases protease activity, we generated a *leuA* deletion mutant, *ΔleuA*. Western blot analysis of actin demonstrated that cellular extracts of *ΔleuA* mycelia cultured during iron starvation and supplemented with 1 mM leucine, which was the lowest leucine concentration supporting *ΔleuA* growth, contain high protease activity (degradation of actin), while supplementation with 3–10 mM leucine decreases protease activity (Fig 4H). In contrast, iron supplementation did not rescue the increased protease activity with 1 mM leucine supplementation (Fig 4I). These data indicate that leucine starvation is the major trigger for increased protease activity in *ΔleuB* during iron starvation. The fact that iron supplementation decreased protease activity in *ΔleuB* but not *ΔleuA* extracts indicates that leucine levels are significantly higher in *ΔleuB* cultured during iron sufficiency compared to iron starvation (Fig 4E and 4I). This is also in agreement with the positive influence of iron on *leuA* transcript levels in both WT and *ΔleuB* strains (Fig 4G).

Taken together, previous reports demonstrating control of *leuA* expression by iron *via* HapX [18] and the current study characterizing regulation of *leuA* expression by LeuB and its connection to protease activity during iron starvation compared to iron sufficiency reveal a regulatory network of HapX and LeuB controlling leucine biosynthesis.

### DNA-binding and the C-terminus are important for LeuB functionality

**Fig 5:**
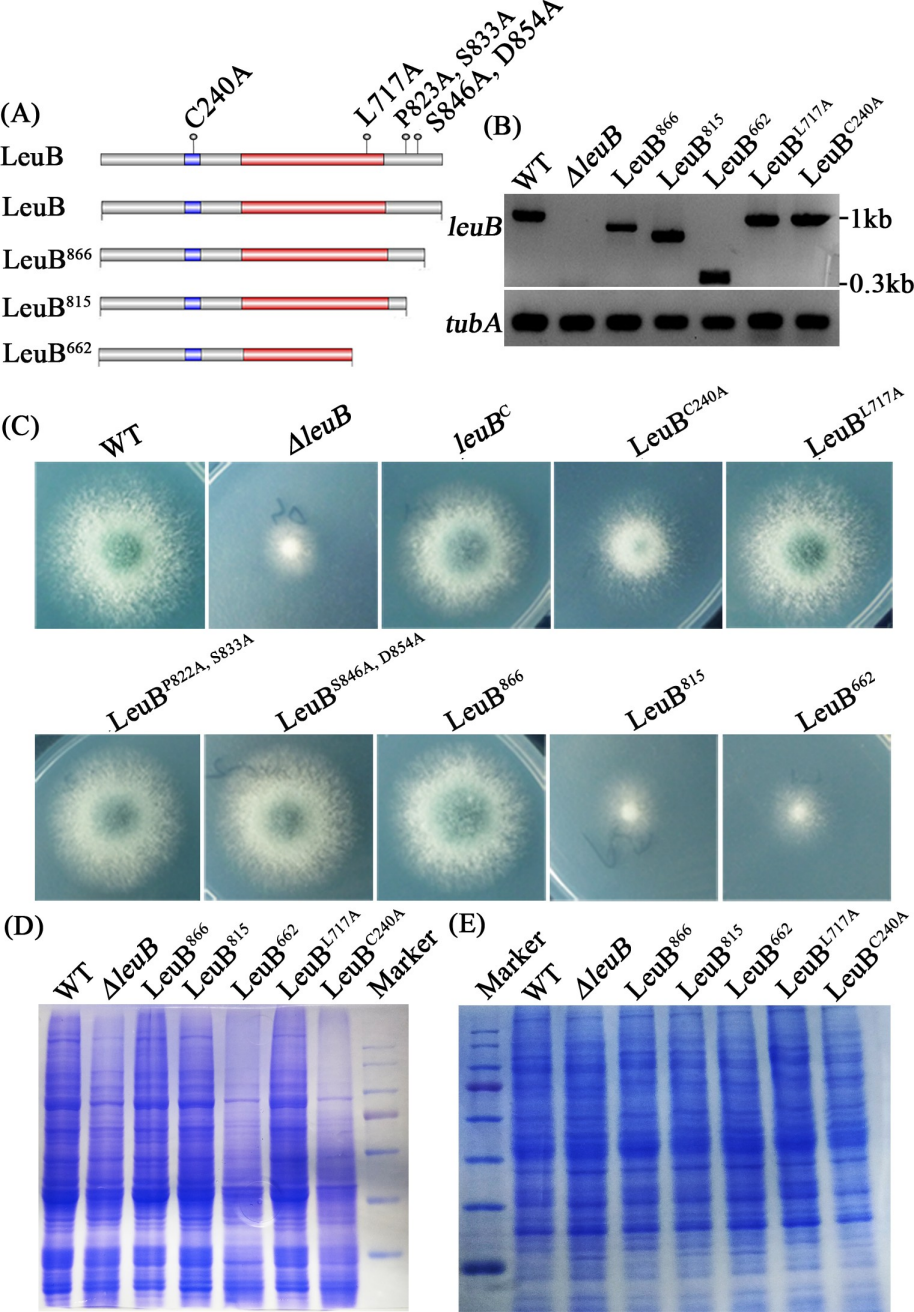
DNA-binding and the C-terminus of LeuB are important for its functionality. (A) Schematic view of LeuB protein domains (DNA binding domain in blue and the potential αIPM-sensing domain in red) displaying introduced site-directed mutations and C-terminal truncations. (B) Verification of expressions of the *leuB* gene variants in the different mutants at the transcriptional level using semi-quantitative RT-PCR analysis. This analysis also illustrates the introduced mutations and truncations for the cDNA amplification using the detection forward primer (RT-LeuB-trunc F) and the reverse primer (RT-LeuB 920 R) designed at the end of the ORF for WT, *ΔleuB*, LeuB^L717A^ and LeuB^C240A^ or at the relative truncated position (RT-LeuB-866 R, RT-LeuB-815 R, RT-LeuB-662 R as shown in S3 Table) for LeuB^866^, LeuB^815^ and LeuB^662^ respectively. The *tubA* gene was used as an internal control. (C) Mutation of the LeuB DNA-binding domain (LeuB^C240A^) as well as truncation of the C-terminal 258 or 105, but not 54, amino acid residues impairs growth on minimal medium. (D) Mutation of the LeuB DNA-binding domain (LeuB^C240A^) as well as truncation of the C-terminal 258 amino acid residues increases proteolytic activity during iron starvation as seen by SDS-PAGE analysis followed by Coomassie blue protein staining using a non-denaturing lysis buffer. (E) Using a denaturing lysis buffer, the increased protease activity observed in (D) is not seen.

In *S*. *cerevisiae*, the C-terminus of the LeuB homolog Leu3 was shown to be crucial for function [19]. Based on these data, a series of C-terminal truncations of LeuB was generated to functionally characterize LeuB protein domains (Fig 5A). Notably, all mutated or truncated LeuB gene variants displayed considerable expression at the transcript level based on semi-quantitative RT-PCR analyses (Fig 5B). Truncation of the C-terminal 54 amino acid residues of LeuB (LeuB^866^) did not significantly affect growth, while truncation of the C-terminal 105 as well as 258 amino acid residues caused a growth phenotype similar to the *ΔleuB* mutant strain (Fig 5C). These data demonstrated that the C-terminus of LeuB is essential for its function. Replacement of cysteine^240^ by alanine (LeuB^C240A^), which is expected to block coordination of zinc in the DNA-binding Zn_2_Cys_6_ domain and consequently DNA-binding, caused a growth defect similar to that caused by lack of LeuB. These data underline the importance of DNA-binding for LeuB function, which is in agreement with its function as transcription factor. In contrast, mutations of some randomly chosen amino acid residues (LeuB^L717A^, LeuB^P823A, S833A^ and LeuB^S846A, D854A^) did not affect the growth pattern. The mutants carrying *leuB* gene variants with truncations or site-directed mutations resulting in growth defects also displayed significantly increased proteolytic activity in cell extracts when grown under iron starvation (Fig 5D and 5E). These data underline the link between LeuB dysfunction and the increased cellular protease activity.

### LeuB directly regulates amino acid biosynthesis, iron metabolism and virulence

**Fig 6:**
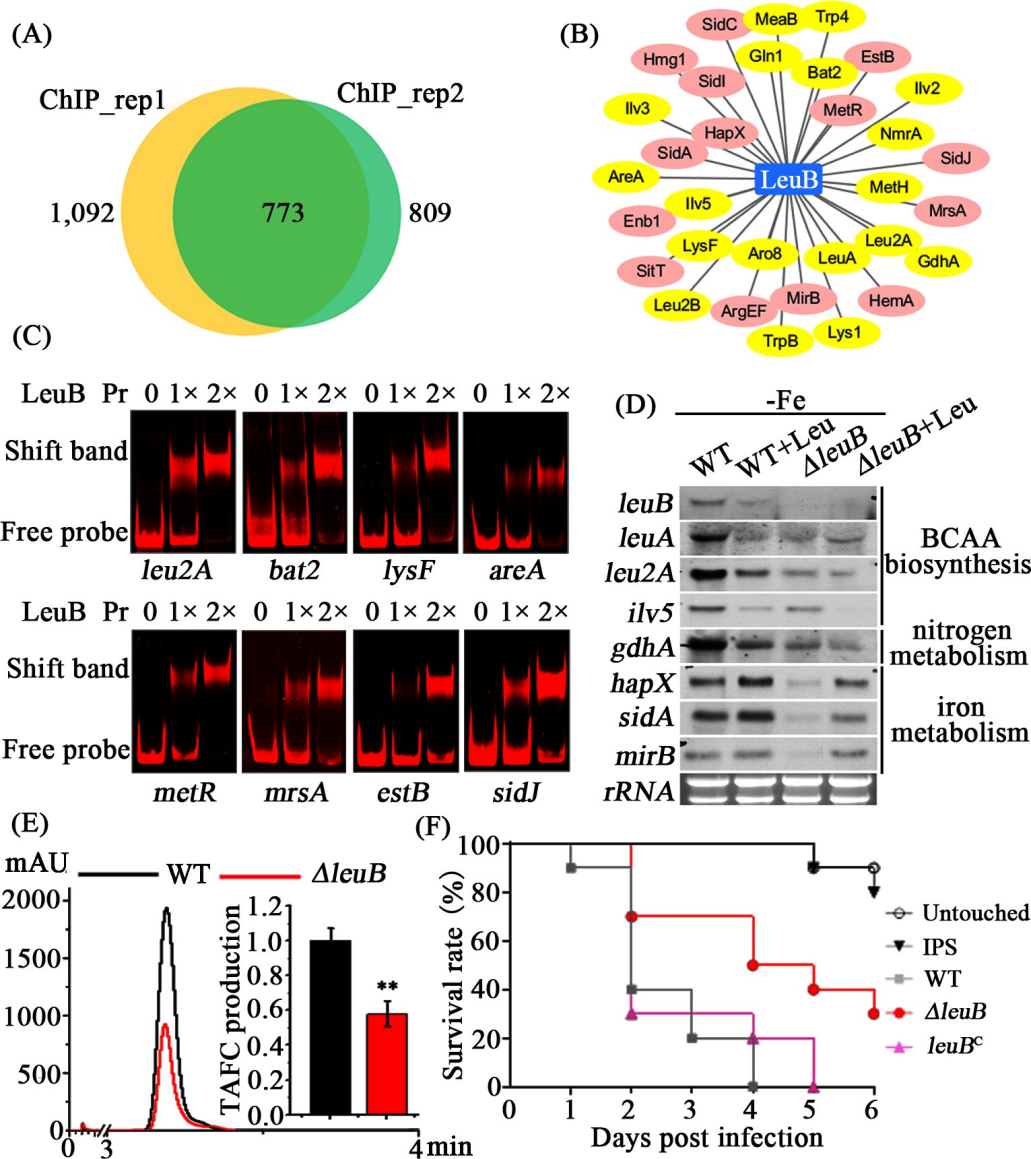
LeuB directly regulates amino acid biosynthesis, nitrogen metabolism and iron metabolism. (A) The number of LeuB target genes identified by two independent ChIP-seq analyses. (B) Genes showing *in vivo* promoter occupation by LeuB revealed by ChIP-seq. Genes labeled in yellow are involved in amino acid and nitrogen metabolism, genes labeled in pink are involved in iron metabolism. (C) EMSA analyses confirming *in vitro* binding of LeuB to promoter fragments of genes involved in amino acid biosynthesis, nitrogen metabolism (*leu2A*, *bat2*, *lysF*, *areA*, *metR*, *mrsA)* and iron metabolism (*estB* and *sidJ*), which showed *in vivo* LeuB promoter occupation in the ChIP-seq analysis. (D) Northern blot analyses demonstrating that during iron starvation, lack of LeuB results in downregulation of genes involved in BCAA biosynthesis, nitrogen metabolism and iron homeostasis. (E) Compared to WT, lack of LeuB results in decreased production of TAFC. TAFC was quantified from supernatants of cultures grown for 24 hours during iron starvation by reversed-phase HPLC analysis; mAU refers to milli-Absorbance Units. TAFC production was normalized to the biomass and the WT production; **represent *p*<0.005. (F) Survival curves of *G*. *mellonella* larvae infected with WT, *ΔleuB*, and *leuB*^C^ strains. Larvae were infected with 1 x 10^7^ conidia, the untouched (untreated) larvae and IPS-injected larvae served as controls. IPS is “Insect Physiological Saline”, the carrier solution for conidia.

To further understand the role of LeuB during iron starvation, chromatin immunoprecipitation followed by massively parallel DNA sequencing (ChIP-seq) was performed to identify genes under direct transcriptional control of LeuB. Therefore, LeuB was C-terminally labeled with the FLAG-tag. The two biological ChIP-seq replicates performed with mycelia cultured during iron starvation identified 1092 and 809 potential target genes, respectively, sharing 773 common targets (Fig 6A and 6B and S1 Dataset). As expected, the ChIP-seq gene set included several genes encoding enzymes involved in BCAA biosynthesis such as *leuA*, *leu2A*, *leu2B*, *bat2*, *ilv2*, *ilv3*, and *ilv5* (the BCAA biosynthetic pathway is shown in S1 Fig.) but also genes involved in biosynthesis of amino acids other than BCAA such as lysine (*lys1*, *lysF*), aromatic amino acids (*aro8*, *trpB*) and proline (*pro3*). Moreover, this analysis indicated that crucial genes of nitrogen and sulfur metabolism are direct LeuB targets, *e*.*g*. nitrogen regulatory protein-encoding *areA*, *meaB* and *nmrA* as well as nitrogen metabolic enzyme-encoding *gdhA* and *glnA* as well as sulfur regulator-encoding *metR*. In agreement with the growth defect of *ΔleuB* during iron starvation, the ChIP-seq analysis also indicated that several genes involved in iron metabolism are direct LeuB targets, including *hapX*, genes involved in siderophore biosynthesis (*sidA*, *sidC*, *sidI*, *hmg1*, *argEF*), siderophore uptake (*mirB*, *enb1*), intracellular siderophore hydrolysis (*sidJ*, *estB*), and mitochondrial iron import (*mrsA*).

In agreement with the ChIP-seq analysis, EMSA and SPR analyses shown above already demonstrated *in vitro* binding of LeuB to the promoters of *leuA*, *gdhA* and *hapX* (Fig 2). Exemplary EMSA analyses further confirmed *in vitro* binding of LeuB to CCGN_4_CGG motif-containing promoter fragments of *leu2A*, *bat2*, *lysF*, *areA*, *metR*, *mrsA*, *estB*, and *sidJ* (Fig 6C), which showed *in vivo* LeuB promoter occupation in the ChIP-seq analysis (Fig 6B).

To further corroborate cross-regulation of BCAA biosynthesis, nitrogen metabolism and iron metabolism by LeuB, we analyzed transcript levels of selected genes in WT and *ΔleuB* strains grown during iron starvation with and without leucine supplementation by Northern analysis (Fig 6D). Transcripts of *leuB* were clearly detectable in WT but not *ΔleuB* confirming the deletion of the *leuB* gene in this strain. In WT, transcript levels of the BCAA genes *leuA*, *leu2A* and *ilv5* were clearly decreased by leucine supplementation indicating transcriptional feed-back inhibition of BCAA biosynthesis by leucine. The transcript levels of these three BCAA genes were significantly decreased in *ΔleuB* compared to WT under growth conditions without leucine supplementation. In agreement with the ChIP data, these results underline that *leuA*, *leu2A* and *ilv5* are directly transcriptionally activated by LeuB during leucine shortage. The decreased *leuA* transcript level in *ΔleuB* compared to WT during iron starvation also matches the Northern blot analysis data shown in Fig 4G. The *gdhA* transcript level was responsive to leucine supplementation and significantly decreased in *ΔleuB* (Fig 6D), again confirming *gdhA* as a direct target of LeuB as shown previously in *A*. *nidulans* and *S*. *cerevisiae* [20,27]. In contrast to BCAA biosynthetic genes and *gdhA*, leucine supplementation did not affect transcript levels of *hapX*, *sidA* (encoding ornithine monooxygenase, which is essential for biosynthesis of extra-and intracellular siderophores) and *mirB* (encoding a transporter for uptake of TAFC) in WT (Fig 6D). However, lack of LeuB significantly decreased expression of these genes, which was partly rescued by leucine supplementation. These data strongly suggest that genes important for regulatory adaptation to iron starvation (*hapX*) and genes involved in SIA (*e*.*g*., *sidA*, *mirB*) are under direct control of LeuB, which explains the growth defect caused by lack of LeuB. In agreement with these data, production of extracellular siderophores (TAFC) by *ΔleuB* reached only 43% of the WT (Fig 6E).

Defective adaptation to iron starvation, *e*.*g*., by lack of HapX or siderophore biosynthesis, was previously shown to attenuate virulence of *A*. *fumigatus* [14,15,17]. Due to the described growth defects, we analyzed the effect of lack of LeuB in the wax moth *Galleria mellonella*. In this insect model, lack of LeuB resulted in a significant higher survival rate of *G*. *mellonella* larvae compared to the WT (p = 0.007) and the complemented strain *leuB*^*C*^ (p = 0.029) over a period of 6 days (Fig 6F). WT and the complemented strain exhibited a similar virulence potential, indicated by survival rates with no statistical difference (p = 0.319). Taken together, these data suggest that LeuB is involved in adaptation to the insect host niche.

## Discussion

Here we functionally characterized the transcription factor LeuB in *A*. *fumigatus*. Orthologs in *S*. *cerevisiae* and *A*. *nidulans* were previously found to transcriptionally regulate BCAA biosynthesis and nitrogen metabolism *via* controlling expression of *gdhA* [20,27]. In agreement with a similar function in *A*. *fumigatus*, lack of LeuB was partially cured by leucine supplementation (Fig 3A and 3B). Moreover, in this study we provide several lines of evidence demonstrating that LeuB cross-regulates BCAA biosynthesis, nitrogen metabolism and iron metabolism: (i) the growth defect caused by lack of LeuB was rescued by supplementation with either leucine or iron (Fig 3B); (ii) similarly, the increased protease activity caused by lack of LeuB was rescued by supplementation with either leucine or iron (Fig 4H and 4E); (iii) genetic interaction studies demonstrated that combining lack of LeuB with lack of the iron-regulatory transcription factor HapX (which is important for adaptation to iron starvation) aggravates the growth defect caused by lack of one of these regulators during iron starvation, while combining lack of LeuB with lack of the iron-regulatory transcription factors SreA (which is important for adaptation to iron excess by repressing iron acquisition) attenuated the growth defect (Fig 3B); (iv) ChIP-seq analysis demonstrated *in vivo* binding to regulatory sequences of genes involved in BCAA biosynthesis, biosynthesis of amino acids apart from BCAA, nitrogen metabolism, and iron metabolism including iron regulation (HapX) and SIA (Fig 6B); (v) EMSA and SPR analyses confirmed high-affinity *in-vitro* binding to CCGN_4_CGG motifs in promoters of genes involved in BCAA biosynthesis and iron metabolism (Figs 2 and 6C), which are phylogenetically conserved in most *Aspergillus* species (Fig 1 and S1 Table); (vi) Northern analysis demonstrated that lack of LeuB causes downregulation of genes involved in BCAA biosynthesis, nitrogen metabolism and iron metabolism (Fig 6D); and, in agreement, (vii) lack of LeuB resulted in decreased production of the siderophore TAFC (Fig 6E).

**Fig 7:**
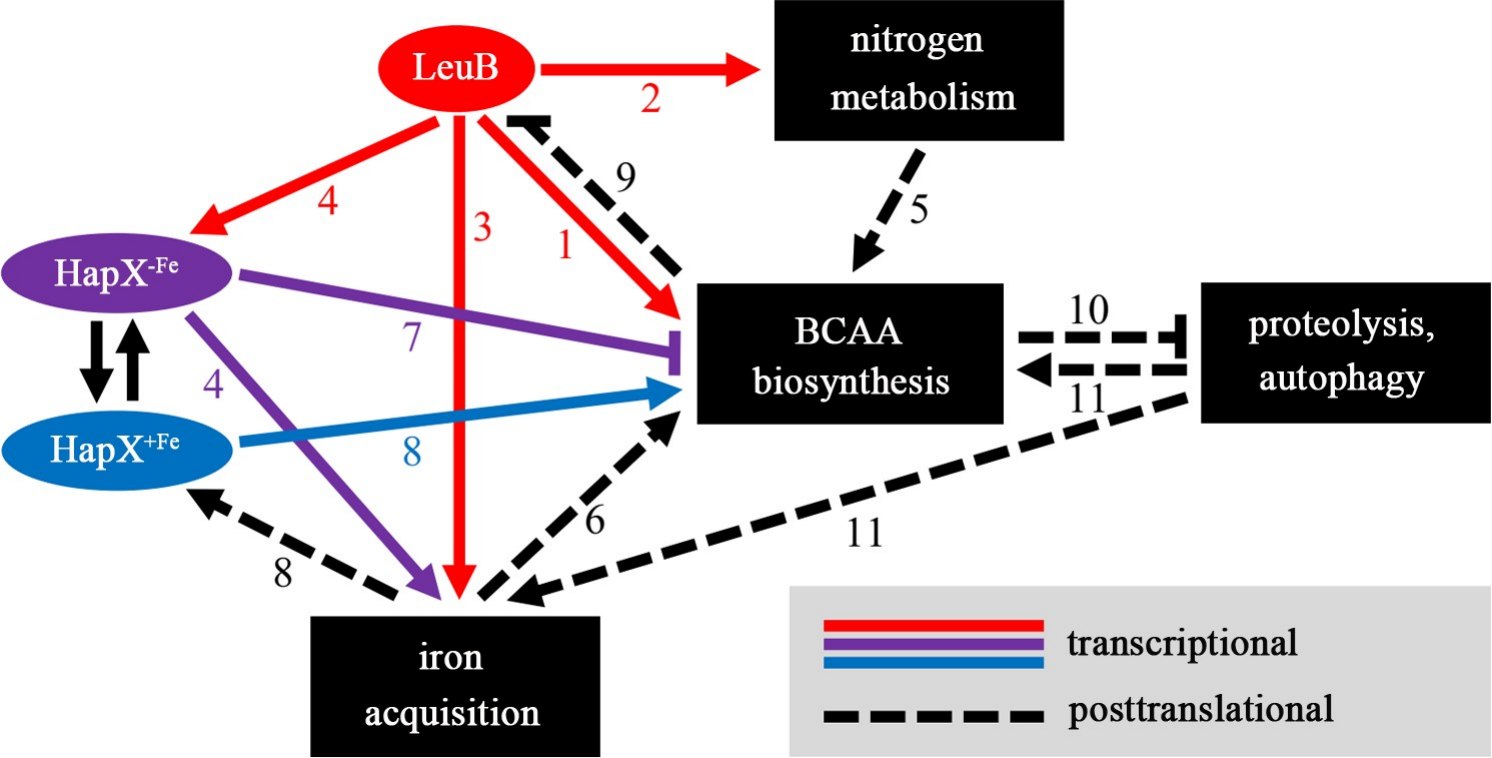
Schematic model of cross-regulation of BCAA biosynthesis, nitrogen metabolism and iron acquisition by LeuB. The regulatory functions are denoted by underlined numbers. LeuB directly transcriptionally activates BCAA biosynthesis (*e*.*g*. *leuA* and *leu2A* genes; labeled 1) nitrogen metabolism (*e*.*g*. *gdhA* gene; labeled 2) and SIA (*e*.*g*. *sidA* and *mirB* genes; labeled 3), the latter also indirectly *via* transcriptional activation of the iron regulator-encoding *hapX* (labeled 4). Cross-regulation of BCAA and nitrogen metabolism by LeuA is most probably based on the nitrogen need for amino acid biosynthesis (labeled 5), while cross-regulation with iron acquisition might be constituted by the iron-dependence of the BCAA biosynthetic enzymes Ilv3 and LeuA (labeled 6). It has previously been shown that during iron starvation HapX (HapX^-Fe^) represses transcription of BCAA biosynthetic genes *ilv3* and *leuA* [17] (labeled 7) and that iron converts HapX into a transcriptional activator (HapX^+Fe^) of these genes (labeled 8), most likely *via* HapX sensing of iron-sulfur clusters [18]. Activity of the *S*. *cerevisiae* LeuB homolog Leu3 has previously been shown to be activated by leucine shortage [19] (labeled 9). During iron starvation, lack of LeuB induces proteolytic activity and autophagy *via* leucine shortage (labeled 10), possibly *via* inactivation of TORC1 [30]. These recycling pathways increase availability of BCAA and iron (labeled 11). Further details are described in the text.

A model summarizing the findings of this study, in the light of previous reports, displaying several regulatory feedback loops in BCAA biosynthesis is depicted in Fig 7. The rational for the LeuB-mediated cross-regulation of BCAA biosynthesis, nitrogen metabolism and iron metabolism is most likely based on the requirement of nitrogen and iron for biosynthesis of BCAA. For example, apart from the general nitrogen-requirement for amino acid biosynthesis, glutamate generated by GdhA is required for the last step in BCAA biosynthesis catalyzed by the transaminases Bat1/2 [19]. The iron dependence of BCAA biosynthesis is based on the requirement of iron-sulfur clusters as prosthetic group for two enzymes, mitochondrial dihydroxyacid dehydratase (Ilv3; in contrast to *S*. *cerevisiae*, which contains only one dihydroxyacid dehydratase-encoding gene, *A*. *fumigatus* encodes three homologs, termed Ilv3A-C [28]) and cytosolic 3-isopropylmalate dehydratase (termed Leu1 in *S*. *cerevisiae* and LeuA in *A*. *fumigatus*) [19]. Consequently, upregulation of leucine biosynthesis increases the need for iron, which we hypothesize to be the rational for the cross-regulation of BCAA biosynthesis and iron acquisition. In other words, the fact that iron starvation hampers leucine production might be employed to indirectly sense iron starvation *via* leucine shortage to upregulate both BCAA biosynthesis and iron acquisition. As iron starvation represses BCAA biosynthesis at the enzyme activity level (lack of prosthetic iron sulfur clusters) and *via* HapX at the regulatory level (HapX represses transcription of *leuA* and *ilv3* during iron starvation [17]), and as BCAA limitation activates LeuB, activation of iron acquisition by LeuB represents a regulatory feedback loop. Remarkably, LeuB transcriptionally activates iron acquisition by direct transcriptional activation of SIA genes (*e*.*g*. *sidA* and *mirB*) as well as indirectly *via* transcriptional activation of the iron regulator-encoding *hapX*. Previous studies revealed another interrelation between BCAA biosynthesis and iron metabolism: during iron starvation, HapX (HapX^-Fe^) represses transcription of *ilv3* and *leuA* together with numerous other “iron-dependent genes” [17], while iron sufficiency converts HapX into a transcriptional activator (HapX^+Fe^) of these genes, most likely *via* HapX sensing of iron-sulfur clusters [18]. Forming another feed-back, activity of the *S*. *cerevisiae* LeuB homolog Leu3 has previously been shown to be posttranslationally activated by leucine shortage *via* the BCAA biosynthesis intermediate α-isoproylmalate (αIPM) [19]. To foster this regulation, the enzyme producing α-isoproylmalate, α-isoproylmalate synthase (termed Leu4/Leu9 in *S*. *cerevisiae* and LeuC in *A*. *fumigatus*, respectively) is inhibited by leucine at the enzyme activity level. However, the intracellular nuclear localization of Leu3 is not affected by leucine or αIPM levels, suggesting that Leu3 binds constitutively to its regulatory sites in *S*. *cerevisiae* [29]. Similarly, the current study revealed that neither leucine nor iron levels affect nuclear localization of LeuB in *A*. *fumigatus* (Fig 3C), suggesting that activation of LeuB may be not affected by trafficking of LeuB probably required for the conformation change of LeuB. In addition, studies of mutants in *A*. *nidulans* with perturbed levels of αIPM indicated that αIPM regulates LeuB [20,27], which is similar to *S*. *cerevisiae*, suggesting evolutionary conservation of this feed-back regulation also existed in *Aspergillus* spp. Previous studies indicated that αIPM-sensing by *S*. *cerevisiae* Leu3 requires the region between amino acid residues 174–773 [19]. This region shows significant similarity in Leu3/LeuB homologs of *S*. *cerevisiae*, *A*. *nidulans* and *A*. *fumigatus* (25% identity/ 43% similarity between *A*. *fumigatus* and *S*. *cerevisiae*; 79% identity/87% similarity between *A*. *fumigatus* and *A*. *nidulans*); an alignment of fungal Leu3/LeuB homologs is shown in S5 Fig). These data strongly suggest that *A*. *fumigatus* LeuB might be also regulated by αIPM.

Furthermore, the current study revealed that lack of LeuB increases cellular proteolytic activity and autophagy *via* leucine shortage during iron starvation but not iron sufficiency. Several lines of evidence suggest that this might be mediated by the “target of rapamycin complex 1 (TORC1)”. TORC1 activates cell proliferation and growth during nutrient availability, while lack of nutrient availability (starvation) blocks TORC1 activity resulting in induction of proteasome activity and autophagy to recycle nutrients [30]. Notably, leucine is one of the major signals activating TORC1, thereby blocking activation of the proteasome and autophagy. These recycling pathways then increase the availability of iron and BCAA. In agreement, autophagy was found to be important for fitness of *A*. *fumigatus* during metal depletion [31].

The fact that lack of LeuB resulted in a similar growth pattern in *A*. *fumigatus* and *A*. *nidulans* indicates that this regulatory circuit is conserved at least in *A*. *nidulans* (S3C Fig). Moreover, the evolutionary conservation of the CCGN_4_CGG motifs in most *Aspergillus* species predicts conservation of this regulatory circuit within this genus. Site-directed mutagenesis and C-terminal truncations of LeuB demonstrated that DNA-binding and the C-terminus are essential for the LeuB functions confirming that LeuB acts indeed as a classical transcription factor (Fig 5A and 5C). Defects in adaptation to iron starvation, *e*.*g*., by lack of HapX or siderophore biosynthesis, have previously been shown to attenuate virulence of *A*. *fumigatus* [14,15,17]. In agreement, lack of LeuB caused attenuation of virulence in the wax moth model (Fig 6F). Notably, inactivation of leucine biosynthesis by deleting the gene encoding Leu1 (the homolog of *A*. *fumigatus* LeuA) was previously shown to attenuate virulence of the basidiomycete yeast *Cryptococcus neoformans* [32]. Moreover, this study demonstrated that leucine shortage leads to increased abundance of two mitochondrial iron-sulfur cluster proteins (aconitase and the iron-sulfur cluster biosynthetic enzyme Nfu1) during iron starvation but not iron sufficiency, while a cytosolic iron-sulfur cluster protein (Fra1), was not affected. However, this study did neither provide a mechanistic explanation for the link between leucine shortage and regulation of mitochondrial iron-sulfur cluster proteins, nor did it show any link to iron acquisition.

Taken together, our work underline that the BCAA biosynthetic pathway is not only structurally important but also represents a central road junction involved in cross-regulation of amino acid biosynthesis, nitrogen metabolism, iron homeostasis and cellular proliferation.

## Material and methods

### Strains, media and culture conditions

*A*. *fumigatus* strains, used in this study, are summarized in S2 Table. Generally, *A*. *fumigatus* strains were grown on minimal medium (MM)[33] containing 1% (w/v) glucose and 70 mM NaNO_3_ as sole carbon and nitrogen sources, respectively. For iron starvation, iron was omitted in the trace element solution; for increased iron starvation, the iron-specific chelator bathophenanthroline disulfonate (BPS) was added in iron depleted media. Supplementation with iron (FeCl_3_) and/or leucine was carried out as described in the Figures. Transformants were screened on media containing 200 μg/ml hygromycin B (Shanghai Sangon Co., China). To analyze the phenotype of the mutants, 2 x 10^3^ conidia were point-inoculated on plates. All plates were incubated at 37°C for two days.

### Gene deletion and reconstitution

All primers used in this study are shown in S3 Table. For the generation of the *leuB* deletion cassette, the fusion PCR technique was used as described previously [34]. Briefly, approximately 1 kb of the upstream and downstream flanking sequences of the *leuB* gene were amplified using the primer pairs LeuB P1/P3 and LeuB P4/P6, respectively. The gene *pyr4* from plasmid pAL5 was amplified with the primers pyr4 F/R and used to restore *pyrG* function in the A1160 WT strain. Next, the three aforementioned PCR products were combined and used as template to generate the *leuB* deletion cassette using the primer pair LeuB P2/P5. This fragment was then used to transform the recipient strain A1160.

To construct the *leuA*, *sreA*, *hapX* and *AnleuB* deletion cassettes, the same strategy was employed except the use of different selection markers. *sreA* and *hapX* were deleted with the hygromycin resistance cassette (*hph)* and *AnleuB* was deleted with *AfpyrG*. For the construction of the *AnleuB* null mutant, the recipient strain used was TN02A7. To generate *ΔleuBΔhapX* and *ΔleuBΔsreA* double mutants in *A*. *fumigatus*, *leuB* was disrupted in the *ΔhapX* or *ΔsreA* background respectively.

To reconstitute *ΔleuB* with a functional copy of the *leuB* or *AnleuB* gene, the following strategy was used. First, the fragment of *leuB* or *AnleuB*, which includes the native promoter, 5’UTR, gene sequence and 3’UTR, was amplified with the primer pairs ComLeuB F/R and ComAnleuB F/R respectively and then subcloned into the pEASY-Blunt Zero Cloning Vector (TransGen Biotech) according to the manufacturer’s directions, yielding the plasmids pEASY-leuB and pEASY-AnleuB. In a next step, the *hph* cassette was amplified using the primer pairs hph-SpeI F/R. pEASY-leuB or pEASY-AnleuB and the resistance cassette were digested with *Spe*I and ligated to integrate the *hph* gene into the plasmids as selection marker. The plasmids were then used to transform the *ΔleuB* strain. Transformation of *A*. *fumigatus* was performed as described previously [34]. Gene deletions were confirmed by diagnostic PCR and Southern blot, as shown exemplary in S2 Fig.

### Protein tagging and fluorescence microscopy analyses

To generate the GFP-labeled LeuB strain (LN09), approximately 1.5 kb upstream sequence of *leuB* referred as fragment 1 (except the termination codon) and downstream sequence of *leuB* referred as fragment 2 (including the termination codon) were amplified using LeuB-gfp P1/P3 and LeuB-gfp P4/P6, respectively. Fragment 3, containing a 5×GA linker, the eGFP sequence and the selection marker *AfpyrG* was amplified from plasmid pFNO3 using the primer pair gfp-pyrG F/R. These three fragments were mixed and employed as template to generate the *leuB-gfp* cassette using the fusion PCR technique with the primers LeuB-gfp P2/P5. After the purification of the *leuB-gfp* cassette, this fragment was used to transform the A1160 strain by homologous replacing original copy of the *leuB* gene to generate the *leuB*(p)::LeuB-GFP strain (LN09) which only had one copy of LeuB. To visualize the cell nucleus of the GFP-labeled LeuB strain (LN09), a nuclear localization sequence labeled RFP plasmid were transformed into GFP-labeled LeuB strain to generate the GFP-labeled LeuB and RFP-labeled nucleus strain (LN10).

For the generation of the GFP-labeled Atg8 strain (LN20), Atg8 was labeled with GFP at the N-terminus under the control of the *A*. *nidulans gpdA* (*AngpdA)* promoter. Briefly, the GFP and Atg8 (without ATG) fragments were amplified with the primer pairs GFP F/R and Atg8 F/R, respectively. The resulting fragments were purified and fused by PCR with the primers GFP F and Atg8 R. This GFP-*atg8* cassette was then subcloned into the *Cla*I site of the pBARGPE-1 vector [35], containing the constitutive *AngpdA* promoter, resulting in the plasmid *gpdA*(p)-GFP-Atg8. Subsequently, the *gpdA*(p)-GFP-Atg8 plasmid was ectopically integrated in the genome of A1160 WT strain to generate the strain expressing GFP-labeled Atg8. In this strain, *leuB* was deleted with the aforementioned construct to receive the *ΔleuB* strain expressing GFP-labeled Atg8 (LN21).

To constitutively express LeuB with a FLAG-tag, the ectopic integration method was used. Briefly, 5×flag sequence was amplified using primers Flag F/R from plasmid pFA6a-5×FLAG-kanMX6 and the DNA sequence of *leuB* without stop codon was amplified with LeuB-Flag F/R. These two fragments were then mixed and employed as template to generate the *leuB-flag* cassette using the primer pair LeuB-Flag F and Flag R. After purification of the PCR products, the fused *leuB-flag* cassette was subcloned into the *Cla*I site of pBARGPE-1, yielding the plasmid OE::LeuB-FLAG, and was then used to transform the A1160 strain to generate the FLAG-labeled LeuB strain (LN11).

To visualize the localization of LeuB-GFP, the LN10 strain (LeuB::GFP, RFP-NLS) was grown on coverslips in 3 ml liquid minimal media with or without iron at 37°C for 18 hours. For iron shift experiments, FeCl_3_ was added to a final concentration of 50 μM and incubated for 1–3 hours. To visualize the localization of LeuB-GFP under the condition with or without leucine, the similar strategy was employed. Images were captured using a Zeiss Axio imager A1 microscope (Zeiss, Jena, Germany) and managed with Adobe Photoshop.

### Site-directed mutagenesis and C-terminal truncation

For site-directed mutagenesis, complementary primers harboring the desired mutation in the center position were designed and synthesized. The plasmid pEASY-leuB used for the complementation of *ΔleuB* was employed as template and amplified with the respective primers, including the desired mutations. The resulting PCR products were digested with *Dpn*I and then transformed into *Escherichia coli*. All plasmids used for site-directed mutagenesis were sequenced to verify the mutation. For C-terminal truncation, reverse primers, which comprise a stop codon used for truncation, were designed and synthesized. The plasmid pEASY-leuB was used as template and the fragments were amplified using the primer pair LeuB-trunc F and the respective primer with the desired truncation. Purified PCR products were then co-transformed into the *ΔleuB* strain.

### Semi-quantitative real-time (qRT)-PCR analysis and TAFC quantification

For Semi-qRT-PCR analysis, total RNA was isolated from the frozen mycelium using TRIzol (Roche) as described in the manufacturer’s manual. The digestion of genomic DNA and synthesis of cDNA was performed using HiScript II Q RT SuperMix for qPCR (+gDNA wiper) kit (Vazyme) as described by the supplier. Primer used for semi-qRT-PCR analysis as labelled in S3 Table.

To analyze the production of TAFC, the *ΔleuB* mutant and WT strains were cultured under iron starvation and the supernatant was separated from the mycelia. Subsequently, the TAFC content of the supernatant was determined by reversed-phase HPLC as described previously [36,37].

### Recombinant LeuB protein production and purification for EMSA

Briefly, two exons of LeuB, which encode the Zn_2_Cys_6_ domain (exon 1 with 126 and exon 2 with 334 amino acid residues), were amplified with the primers Ex-LeuB P1/P2 and Ex-LeuB P3/P4 respectively and then fused using the fusion PCR technique with the primers Ex-LeuB P1/P4. The fused cassette, which contains a 6×His tag at the 5’ end, was cloned into the *Nde*I and *Eco*RI site of pET-30a(+). The resulting plasmid was then used for transformation of *E*. *coli* BL21(DE3). The *E*. *coli* cells were cultured in lysogeny broth (LB) medium to an optical density of 0.8 at 37°C measured at 600 nm (OD600) and subsequently induced by 1 mM IPTG at 16°C for 12h. Protein purification was performed as previously described using Ni-NTA agarose [38].

### Electrophoretic mobility shift assay (EMSA)

The electrophoretic mobility shift assay was performed as described previously [39]. Cy5 labeled DNA probes were prepared as followed. A DNA fragment of the promoter region of different genes containing the conserved CCGN_4_CGG motif was amplified by PCR using the respective primer pairs (EMSA-gene name F/R). Forward and reverse primers were labeled with an oligonucleotide, refers as primer pEMSA. The purified PCR product was then employed as template to generate the Cy5 labeled DNA probe using the Cy5 labeled primer pEMSA. For site-directed mutagenesis of the *hapX* probe, extra complementary primers including the desired mutation were designed and named EMSA-MuhapX F/R. Fragments that contain half of the probe sequence were amplified using MuEMSA-hapX F/EMSA-hapX R and MuEMSA-hapX R and EMSA-hapX F, respectively. The purified fragments were then fused using the primers EMSA-hapX F/R to generate the template for the *hapX* DNA probe with site-directed mutagenesis. For nonspecific competitor or cold probe, 1 μg salmon sperm DNA or a 100-fold non-labeled DNA probe was added. The Cy5-labeled probes were detected with Odyssey machine.

### Protein expression and purification of LeuB for SPR analysis

To reduce unspecific binding of the LeuB protein to the SPR-matrix, a cDNA fragment encoding only the DNA binding domain (DBD) of *A*. *fumigatus* LeuB was subcloned into the pET-29a vector (Novagen, Germany). The LeuB^M50-125YS^ protein was produced by autoinduction in *E*. *coli* BL21 (DE3) cells grown at 25°C in 1 l Overnight Express Instant TB Medium (Novagen, Germany) in the presence of 1 mM Zn(OAc)_2_ and kanamycin. 25.8 grams wet cells were collected by centrifugation, resuspended in 200 ml lysis buffer (20 mM HEPES, 150 mM NaCl, 10 μM Zn(OAc)_2_, 5 mM β-mercaptoethanol, 1 mM AEBSF, pH 7.5) and disrupted using an Emulsiflex C5 high pressure homogenizer (Avestin, Germany). The cleared cellular extract was adjusted to pH 7.5, loaded on a SP Sepharose HP column (GE Healthcare, Germany) and eluted with a salt gradient up to 1 M NaCl. The LeuB DBD containing fraction was adjusted to 150 mM NaCl and loaded on a Cellufine Sulfate column (Millipore, Germany), which was previously equilibrated with 20 mM HEPES, 150 mM NaCl, 10 μM Zn(OAc)_2_, 5 mM β-mercaptoethanol, pH 7.5, followed by elution with a salt gradient up to 1 M NaCl. The peak fractions were concentrated by ammonium sulfate precipitation and redissolved in 20 mM HEPES, 150 mM NaCl, 10 μM Zn(OAc)_2_, 5 mM β-mercaptoethanol, pH 7.5. The LeuB DBD was then purified by size exclusion chromatography on a Superdex 75 prep grade column (GE Healthcare) using a 20 mM HEPES, 150 mM NaCl, 10 μM Zn(OAc)_2_, pH 7.5 containing running buffer. The protein was stored in 50% v/v glycerol at -20°C. The purification of LeuB for SPR analysis is shown in S6 Fig.

### Surface plasmon resonance analysis

Real-time analyses were performed on a Biacore T200 system (GE Healthcare) at 25°C. DNA duplexes were produced by annealing complementary 16 bp oligonucleotides using a 5-fold molar excess of the non-biotinylated oligonucleotide. The dsDNA was injected on flow cells of a streptavidin (Sigma)-coated CM3 sensor chip at a flow rate of 10 μl/min until the calculated amount of DNA had been bound that gives a 100 RU maximum LeuB DBD binding capacity. LeuB DBD samples containing 30 μg/ml poly(dAdT) were injected in running buffer (10 mM HEPES pH 7.4, containing 150 mM NaCl, 0.005% (v/v) surfactant P20, 5 mM β-mercaptoethanol and 10 μM ZnCl_2_) at concentrations from 6.25 to 400 nM. Sample injection and dissociation times were set to 100 and 200 seconds at a flow rate of 30 μl/min. Refractive index errors due to bulk solvent effects were corrected with responses from DNA-free flow cell 1 as well as subtracting blank injections. Kinetic raw data were processed and globally fitted with Scrubber 2.0c (BioLogic Software) using a 1:1 interaction model including a mass transport term.

### Protein extraction and Western blotting

For whole-cell lysate extraction, mycelia were ground with liquid nitrogen and two buffers, a mild, non-denaturing lysis buffer (50 mM HEPES pH 7.4, 137 mM KCl, 10% glycerol containing, 1% Triton X-100, 1 mM EDTA, 1 μg/ml pepstatin A, 1 μg/ml leupeptin and 1 mM PMSF) and the alkaline lysis buffer (0.2 M NaOH, 0.2% β-mercaptoethanol). For mild, non-denaturing lysis buffer mediated protein isolation, samples were incubated on ice and vortexed for 30 s every 5 min for three times. Cell debris was removed by centrifugation at 13, 000×g and 4°C for 10 min. The protein concentration in the supernatant was measured by Bio-Rad protein assay kit. For alkaline lysis buffer mediated protein isolation, the following strategy was used. Briefly, 20 mg of powdered mycelium were re-suspended in 1 ml lysis buffer. 75 μl of trichloroacetic acid (TCA) were added, the samples were vortexed and incubated on ice for 10 min. After centrifugation at 13,000×g, for 5 min at 4°C, the supernatants were removed and the pellets were heated up to 95°C and vortexed in 100 μl of 1 M Tris and 100 μl of 2×SDS protein sample buffer until complete dissolution. For Western blot analysis, GFP and actin were detected with the anti-GFP mouse monoclonal antibody (Roche, Cat. No. 11 814 460 001), anti-actin antibody (ICN Biomedicals Inc., clone C4), respectively. The detailed Western blotting procedure was described previously [40].

### *G*. *mellonella* virulence assay

Virulence assays in *G*. *mellonella* were carried out according to Fallon et al. [41]. *G*. *mellonella* larvae (SAGIP, Italy) were kept in the dark at 18°C before use. Larvae, in groups of 20, were injected through one of the hind pro-legs with 20 μl of IPS (“Insect Physiological Saline”: 150 mM NaCl, 5 mM KCl, 10 mM EDTA, and 30 mM sodium citrate in 0.1 M Tris–HCl, pH 6.9) containing 1 x 10^7^ conidia of the respective strain. Untreated larvae and larvae injected with 20 μl of IPS served as controls. Larvae were incubated at 30°C in the dark and monitored daily up to 6 days. Significance of survival data was evaluated by using Kaplan-Meier survival curves, analyzed with the log-rank (Mantel Cox) test utilizing GraphPad Prism software. Differences were considered significant at *p* values < 0.05.

### Protease activity measurement

For quantification of chymotrypsin-like protease activity in cell extracts, proteins of the WT and *ΔleuB* strains were isolated with the aforementioned non-denaturing lysis buffer from cultures grown under iron starvation. Protein concentration was measured with the Bio-Rad protein assay kit. To determinate the chymotrypsin-like activity, the degradation of the fluorogenic peptide succinyl-Leu-Leu-Val-Try-7-amido-4- methylcoumarin (Suc LLVY-AMC; 0.167 mg/ml in 100 mM Tris–HCl, pH7.4; excitation 360 nm, emission 460 nm) was detected as described previously [42,43].

### ChIP-seq analysis

For ChIP-seq, the FLAG-labeled LeuB strain (LN11) was cultured under iron starvation for 24 h and cross-linked by addition of 1% formaldehyde for 10 min under shaking (100 rpm) at 37°C. Crosslinking was stopped by adding glycine to a final concentration of 0.125 M and incubated at room temperature for 5 minutes under shaking. Mycelia were washed with pre-cold PBS and collected using vacuum filtration. Subsequently the collected mycelia were frozen with liquid nitrogen. DNA sonication, chromatin immunoprecipitation, DNA purification and ChIP-seq were performed by Bio-tech & Consult (Shanghai) Co. LTD. Peaks of the ChIP-Seq were called using Model-based Analysis for ChIP-Sequencing (MACS2, version 2.1.1.20160309). Peaks calling were done with the ChIP-seq samples and input samples with a q-value cutoff of 5.00e-02. The obtained data were further analyzed to screen the putative target genes that contain the CCGN4CGG motif in the predicted promoter or 5’UTR.

### Northern blot analysis

The WT strain with and without leucine supplementation (5 mM) and the *ΔleuB* strain with leucine supplementation (5 mM) were cultured for 16 hours during iron starvation at 37°C. To compensate for the reduced growth rate and to yield the same biomass formation, the *ΔleuB* strain was cultured for 18 hours without leucine supplementation. RNA was isolated from the harvested mycelia using TRI Reagent (Sigma-Aldrich, Vienna, Austria) according to the manufacturer's description. For Northern blot analysis, 10 μg of total RNA (2.5 μg for the detection of *gdhA* RNA levels) were loaded on a 2.2 M formaldehyde agarose gel for electrophoresis and subsequently blotted onto an Amersham Hybond N membrane (GE Healthcare, Vienna, Austria). RNA levels were detected with PCR amplified DIG-labeled probes. Primers used for amplification of the hybridization probes are listed in S3 Table.

## Supporting information

S1 Fig The BCAA biosynthetic pathway in *S*. *cerevisiae* [19]. Enzymes are boxed in grey or in blue if transcriptionally activated by Leu3. Enzymes termed differently in *A*. *fumigatus* are boxed in black. Feed-back inhibition of Leu4/9 enzymatic activity by leucine is shown in green. Posttranslational activation of Leu3 by the leucine biosynthesis intermediate α-isopropylmalate (in blue) is shown as dashed blue arrow. Enzymes requiring iron-sulfur clusters are framed in red.
(TIF)

S2 Fig Confirmation of deletion of *leuA* and *leuB* by diagnostic PCR and Southern blot analysis. (A) For *leuA*/*leuB*, PCR-amplification of the 5´-flanking region (“left arm”, using primers LeuA/B P1+De-pyr4 R, 1638/986 bp) and the 3'-flanking region (“right arm” using primers LeuA/B P6+De-pyr4 F, 2188/1610 bp) was used to verify replacement of *leuA*/*leuB* by the *pyr4* marker. Lack of amplification of the *leuA*/*leuB* coding sequence (“gene self” using primers LeuA/B S1/2, 1262/493 bp) was used to confirm deletion. (B) Southern blot analysis (right) with schematic view (left) demonstrating replacement of *leuB* by the *N*. *crassa pyr4* gene.
(TIF)

S3 Fig Phenotype analysis of *ΔleuB* and *ΔAnleuB*. (A) Biomass production of WT, *ΔleuB*, *leuB*^C^, *ΔhapX*, *ΔsreA*, *ΔleuBΔhapX* and *ΔleuBΔsreA* strains in liquid culture conditions with or without supplementation with iron (FeCl_3_, 50 μM) or leucine (2 mM). 1×10^8^ conidia of the indicated strain were cultured in 100 ml liquid media at 37°C for 24h; * represents *p*<0.05, **represents *p*<0.005. (B) Phenotype analysis of *ΔleuB* on solid minimal medium under iron starvation with supplementation with 5 mM Mg^2+^, Ca^2+^ and Mn^2+^, respectively. (C) Phenotype analysis of *ΔAnleuB* on solid minimal medium with respect to iron availability.
(TIF)

S4 Fig Lack of LeuB results in significantly increased accumulation of N-terminally GFP-tagged Atg8 in vacuoles or autophagosomes. Fungal strains were grown in duplicates during iron starvation. Epi-fluorescence analysis is denoted GFP-Atg8; GFP-Atg8 accumulation is marked by white arrows. Images with differential interference contrast microscopy is denoted DIC; Scale bar = 10 μm.
(TIF)

S5 Fig Alignment of fungal Leu3/LeuB homologs. AfuLeuB, *A*. *fumigatus*; AnLeuB, *A*. *nidulans*, NcLeuB, *Neurospora crassa*, CaLeu3, *Candida albicans*, CgLeu3, *Candida glabrata*, ScLeu3, *S*. *cerevisiae*.
(TIF)

S6 Fig Characterization of the recombinant LeuB polypeptide used for SPR analysis. (A) Amino acid sequence of the LeuB DNA-binding domain. Conserved cysteine residues are colored in red. (B) SDS-PAGE analysis of recombinant LeuB purification: Lane 1, SP sepharose pool; lane 2, SP sepharose unbound; lane 3, SP sepharose pool; lane 4, cellufine sulfate unbound; lane 5; cellufine sulfate pool; lane 6, ammonium sulfate precipitation; lane 7, size exclusion chromatography pool.
(TIF)

S1 Table Promoters of the iron regulatory gene *hapX*, the nitrogen metabolic gene *gdhA* and the BCAA biosynthetic genes *leuA*, *ilv5*, *leuC*, and *leu2A* contain phylogenetically conserved CCGN_4_CCG motifs. Conserved motifs in 20 *Aspergillus* spp. downloaded from AspGD (http://www.aspergillusgenome.org) were identified by MEME analysis (http://meme-suite.org/tools/meme) within 1-kb 5’-upstream regions.
(DOCX)

S2 Table *A*. *fumigatus* strains used in this study. (DOCX)

S3 Table Primers used in this study. (DOCX)

S1 Dataset ChIP-Seq data of LeuB. (XLS)
