# Supplementary figures and images for: The Zn2Cys6-type transcription factor LeuB cross-links regulation of leucine biosynthesis and iron acquisition in Aspergillus fumigatus
Source: PLoS Genet. 2018 Oct 26;14(10):e1007762. doi: 10.1371/journal.pgen.1007762 (PMC6221358; doi:10.1371/journal.pgen.1007762)

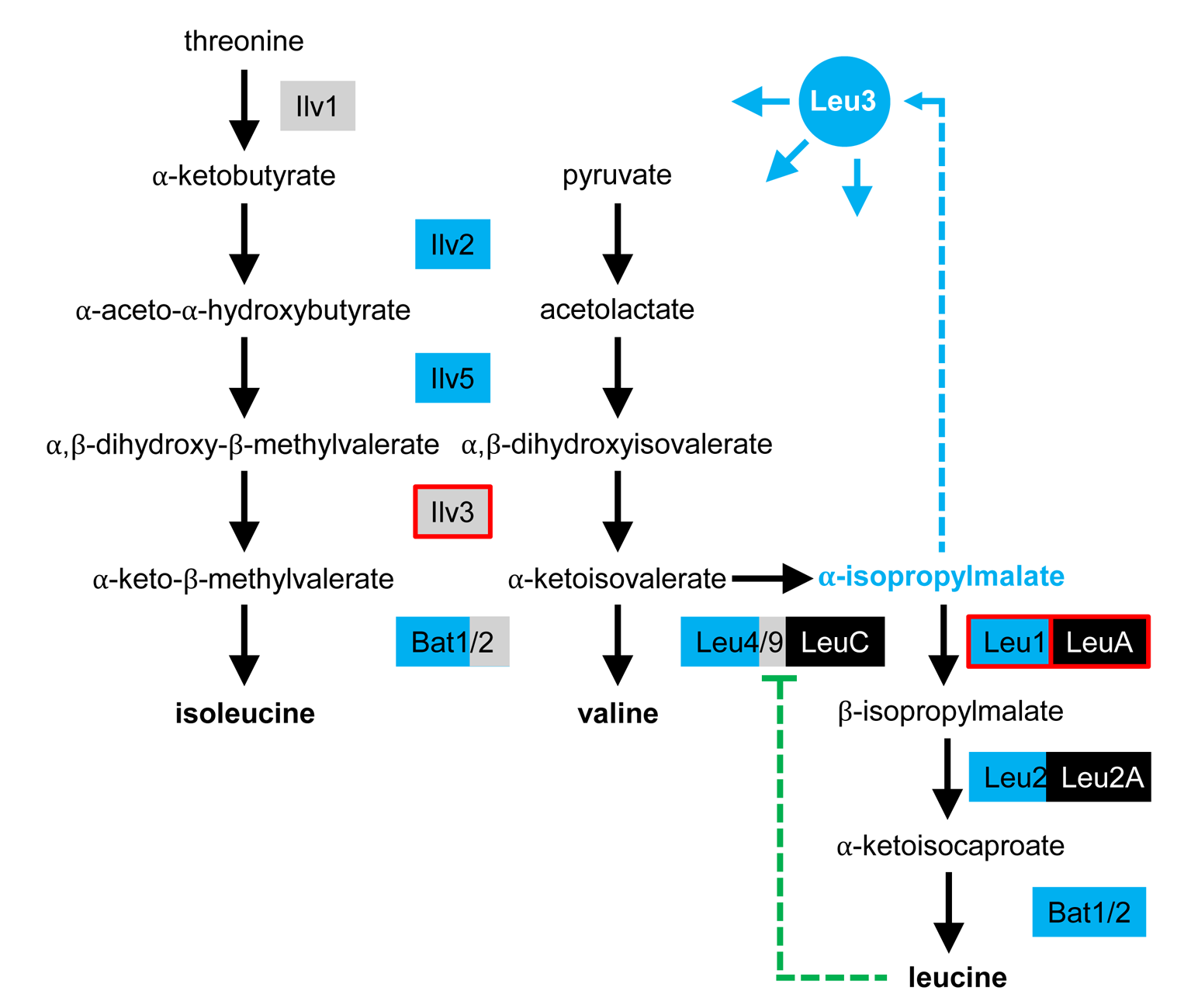

Supplement: S1 Fig — Enzymes are boxed in grey or in blue if transcriptionally activated by Leu3. Enzymes termed differently in A. fumigatus are boxed in black. Feed-back inhibition of Leu4/9 enzymatic activity by leucine is shown in green. Posttranslational activation of Leu3 by the leucine biosynthesis intermediate α-isopropylmalate (in blue) is shown as dashed blue arrow. Enzymes requiring iron-sulfur clusters are framed in red. (TIF) [file pgen.1007762.s001.tif]

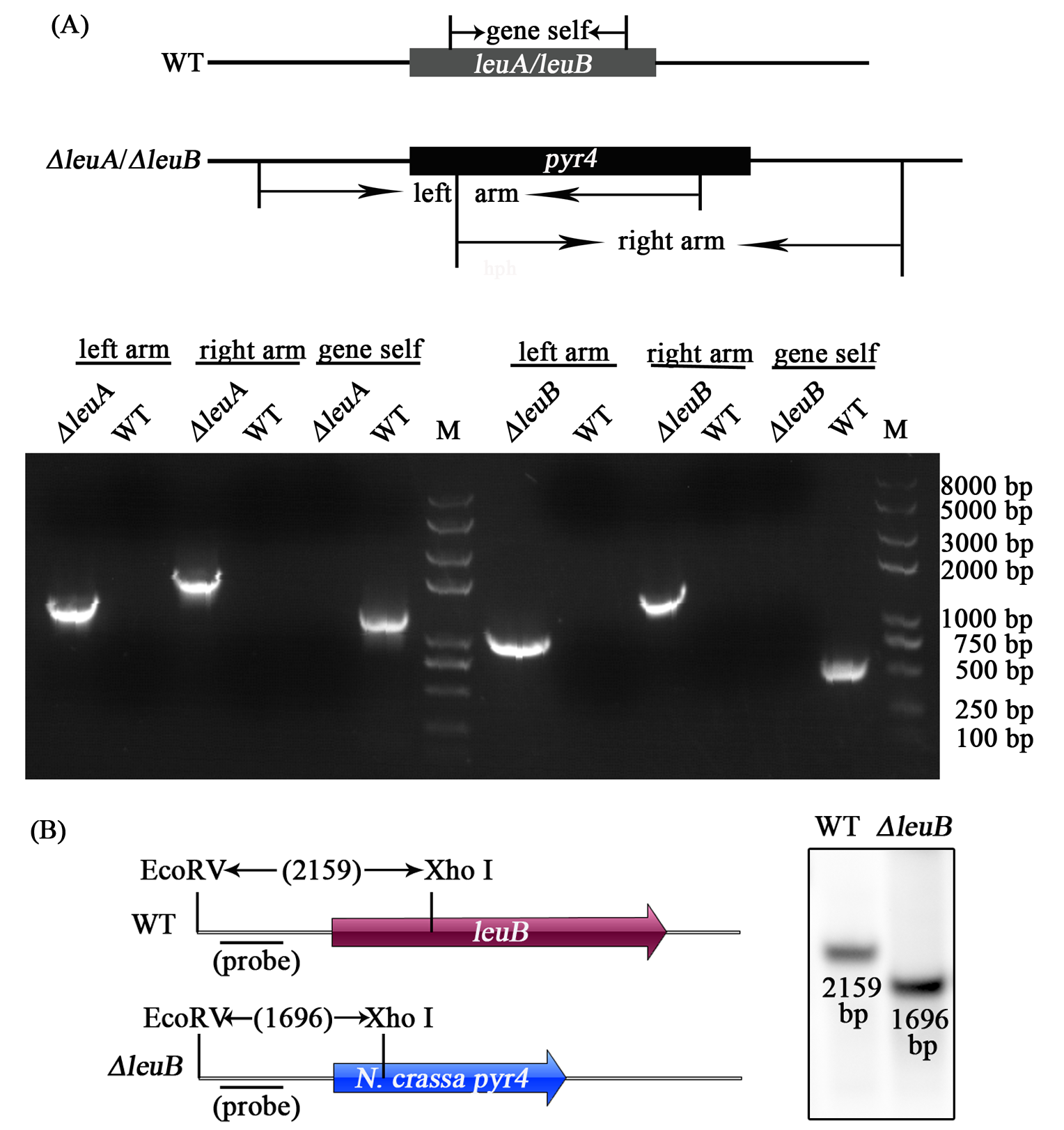

Supplement: S2 Fig — (A) For leuA/leuB, PCR-amplification of the 5´-flanking region (“left arm”, using primers LeuA/B P1+De-pyr4 R, 1638/986 bp) and the 3'-flanking region (“right arm” using primers LeuA/B P6+De-pyr4 F, 2188/1610 bp) was used to verify replacement of leuA/leuB by the pyr4 marker. Lack of amplification of the leuA/leuB coding sequence (“gene self” using primers LeuA/B S1/2, 1262/493 bp) was used to confirm deletion. (B) Southern blot analysis (right) with schematic view (left) demonstrating replacement of leuB by the N. crassa pyr4 gene. (TIF) [file pgen.1007762.s002.tif]

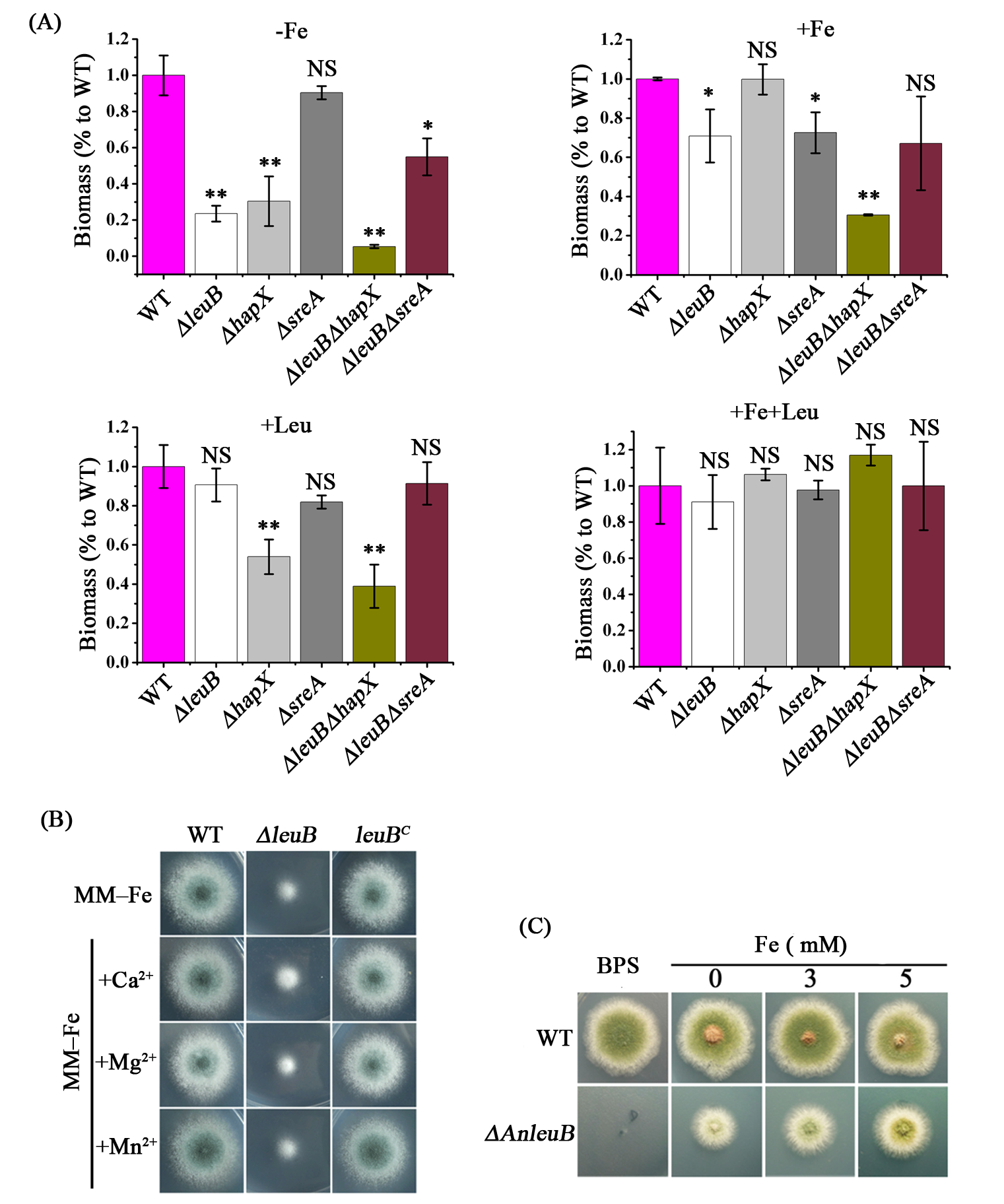

Supplement: S3 Fig — (A) Biomass production of WT, ΔleuB, leuBC, ΔhapX, ΔsreA, ΔleuBΔhapX and ΔleuBΔsreA strains in liquid culture conditions with or without supplementation with iron (FeCl3, 50 μM) or leucine (2 mM). 1×10^8^ conidia of the indicated strain were cultured in 100 ml liquid media at 37°C for 24h; * represents p<0.05, **represents p<0.005. (B) Phenotype analysis of ΔleuB on solid minimal medium under iron starvation with supplementation with 5 mM Mg2+, Ca2+ and Mn2+, respectively. (C) Phenotype analysis of ΔAnleuB on solid minimal medium with respect to iron availability. (TIF) [file pgen.1007762.s003.tif]

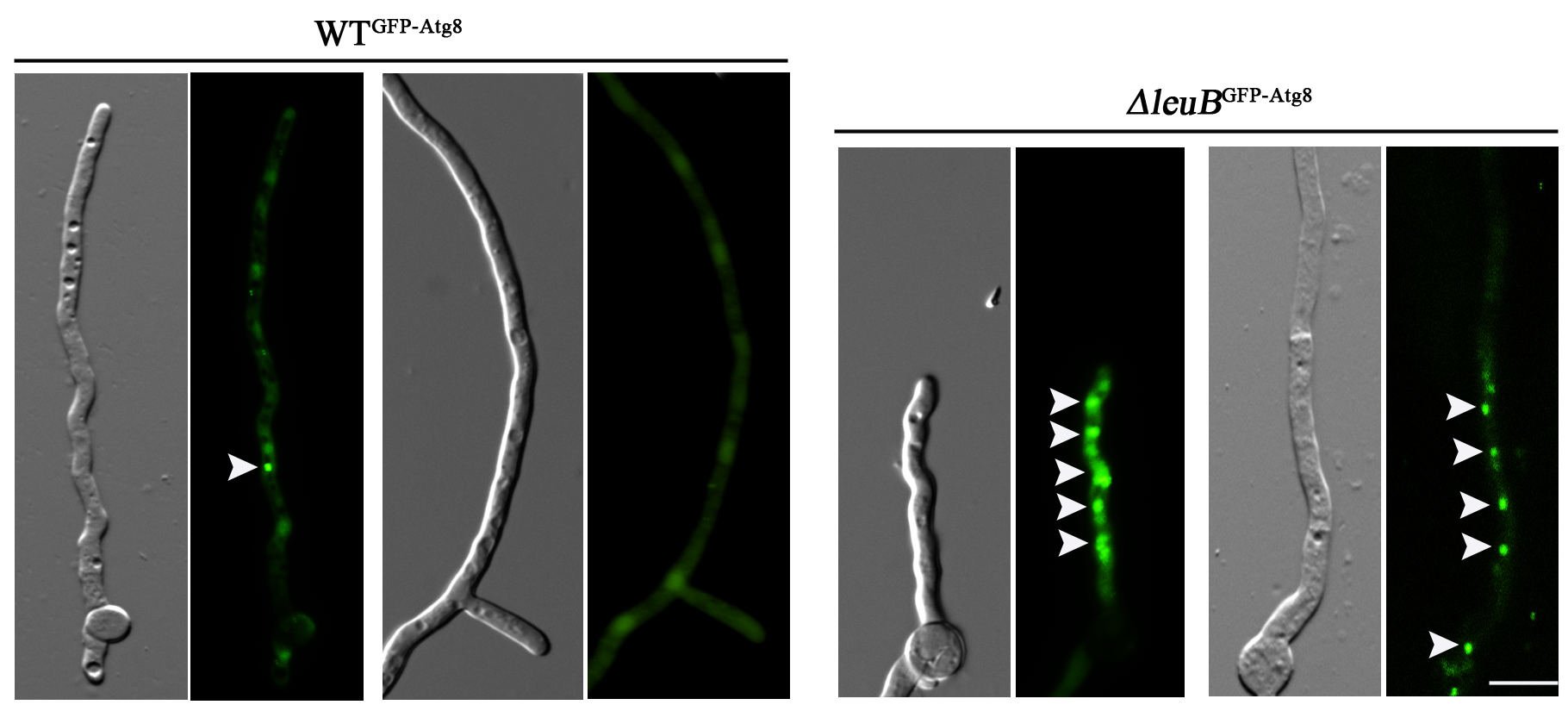

Supplement: S4 Fig — Fungal strains were grown in duplicates during iron starvation. Epi-fluorescence analysis is denoted GFP-Atg8; GFP-Atg8 accumulation is marked by white arrows. Images with differential interference contrast microscopy is denoted DIC; Scale bar = 10 μm. (TIF) [file pgen.1007762.s004.tif]

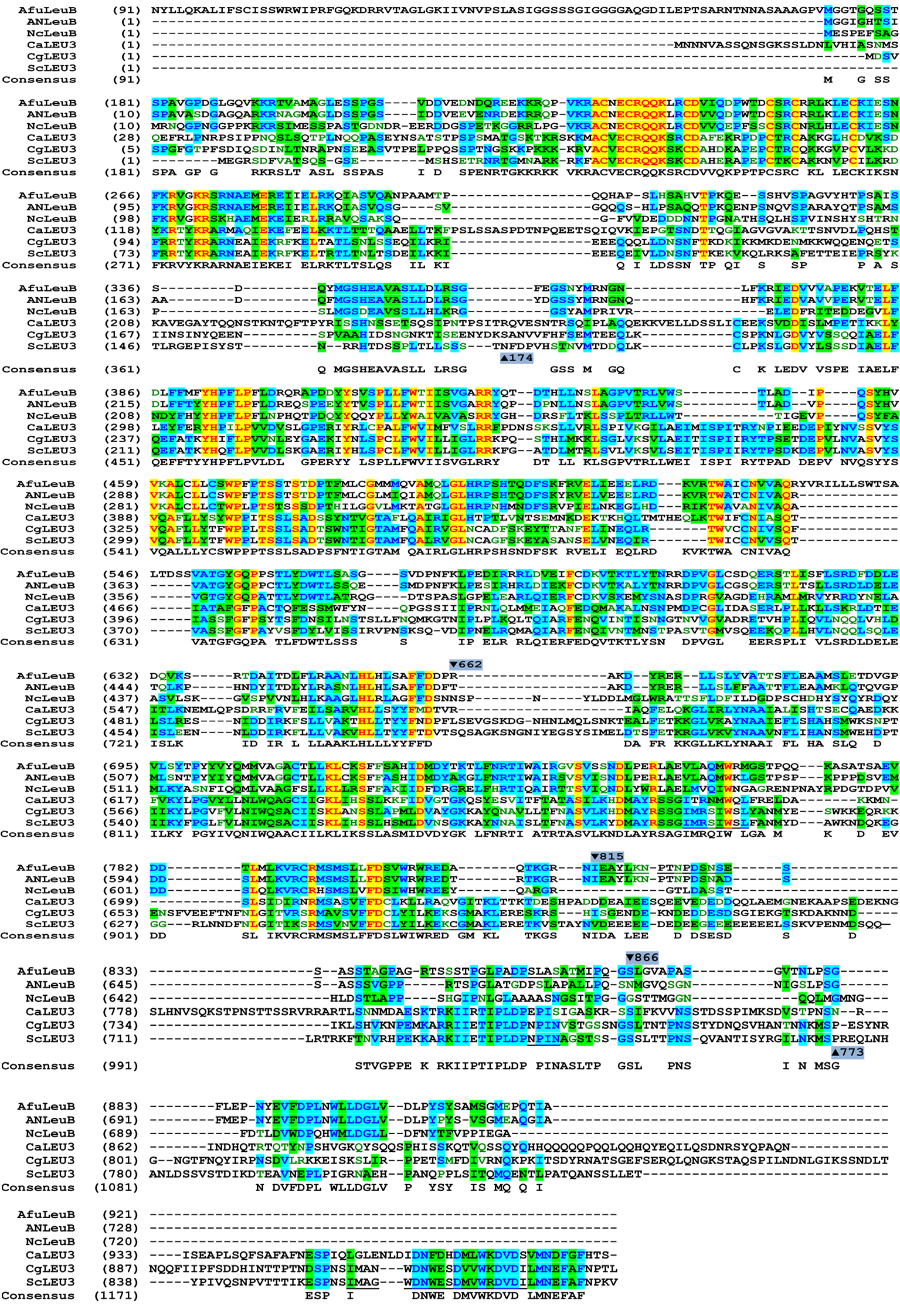

Supplement: S5 Fig — AfuLeuB, A. fumigatus; AnLeuB, A. nidulans, NcLeuB, Neurospora crassa, CaLeu3, Candida albicans, CgLeu3, Candida glabrata, ScLeu3, S. cerevisiae. (TIF) [file pgen.1007762.s005.tif]

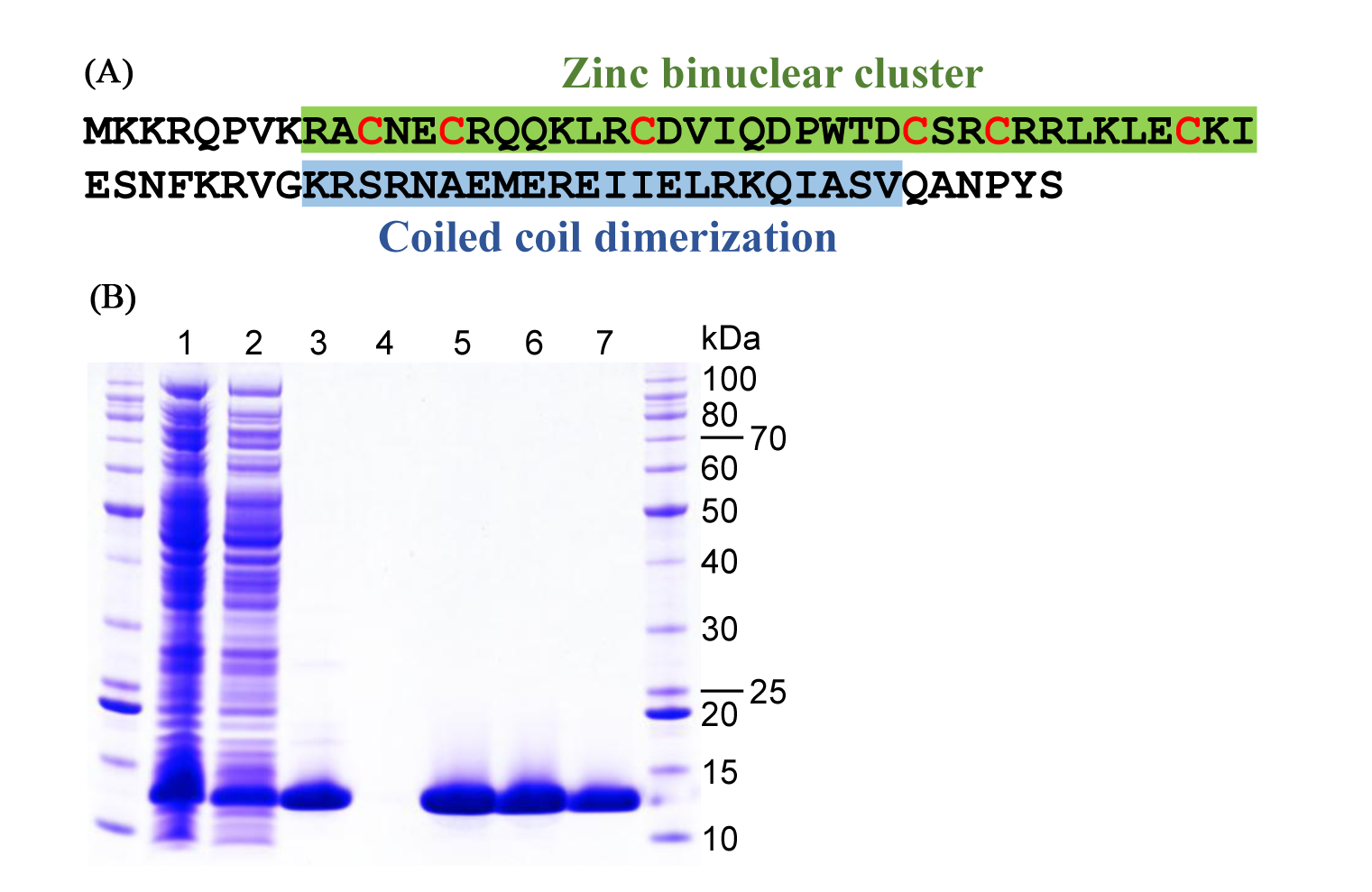

Supplement: S6 Fig — (A) Amino acid sequence of the LeuB DNA-binding domain. Conserved cysteine residues are colored in red. (B) SDS-PAGE analysis of recombinant LeuB purification: Lane 1, SP sepharose pool; lane 2, SP sepharose unbound; lane 3, SP sepharose pool; lane 4, cellufine sulfate unbound; lane 5; cellufine sulfate pool; lane 6, ammonium sulfate precipitation; lane 7, size exclusion chromatography pool. (TIF) [file pgen.1007762.s006.tif]
