# Supplementary material for: The Zn2Cys6-type transcription factor LeuB cross-links regulation of leucine biosynthesis and iron acquisition in Aspergillus fumigatus
Source: PLoS Genet. 2018 Oct 26;14(10):e1007762. doi: 10.1371/journal.pgen.1007762 (PMC6221358; doi:10.1371/journal.pgen.1007762)
Supplement: S1 Table — Conserved motifs in 20 Aspergillus spp. downloaded from AspGD (http://www.aspergillusgenome.org) were identified by MEME analysis (http://meme-suite.org/tools/meme) within 1-kb 5’-upstream regions. (DOCX) [file pgen.1007762.s007.docx]

**S1 Table. Promoters of the iron regulatory gene *hapX*, the nitrogen metabolic gene *gdhA* and the BCAA biosynthetic genes *leuA, ilv5, leuC*, and *leu2A* contain phylogenetically conserved CCGN_4_CCG motifs.** Conserved motifs in 20 *Aspergillus* spp. downloaded from AspGD were identified by MEME analysis (http://meme-suite.org/tools/meme) within 1-kb 5’-upstream regions.

| Gene | *A. fumigatus* gene ID | Motif | Sites | AFU 5´ position | Consensus motif |
| --- | --- | --- | --- | --- | --- |
| *hapX* | AFUA_5G03920  AFUB_052420 | 4 | 20 | -485(+) | 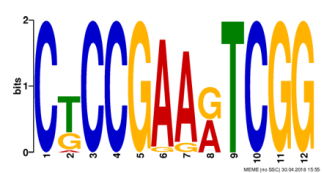 |
| *leuA* | AFUA_2G11260  AFUB_027020 | 2 | 20 | -245(+) | 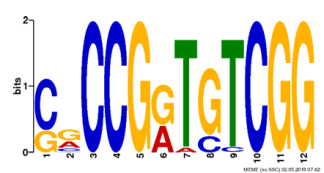 |
| *ilv5* | AFUA_3G14490  AFUB_034740 | 1 | 20 | -224(+) | 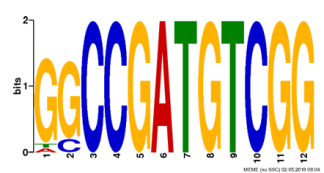 |
| *leuC* | AFUA_1G15000  AFUB_014560 | 1 | 18 | -296(+) | 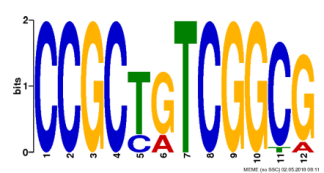 |
| *leu2A* | AFUA_1G15780  AFUB_015310 | 1 | 20 | -125(+) | 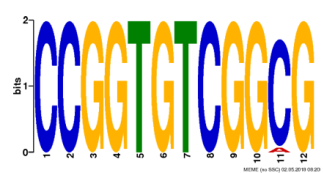 |
| *gdhA* | AFUA_4G06620  AFUB_063700 | 1 | 20 | -232(+) | 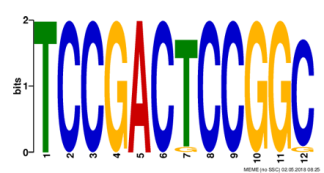 |
