# Supplementary material for: The Zn2Cys6-type transcription factor LeuB cross-links regulation of leucine biosynthesis and iron acquisition in Aspergillus fumigatus
Source: PLoS Genet. 2018 Oct 26;14(10):e1007762. doi: 10.1371/journal.pgen.1007762 (PMC6221358; doi:10.1371/journal.pgen.1007762)
Supplement: S2 Table — (DOCX) [file pgen.1007762.s008.docx]

**S2 Table. *A. fumigatus* strains used in this study**

| Strain name | Genotype | Reference |
| --- | --- | --- |
| A1160 | *Δaku80*, *pyrG1* | FGSC |
| TN02A7 | *pyrG89*, *pyroA4*, *nkuA::argB2*, *riboB2*, *veA1* | FGSC |
| WT | A1160, *pyrG* | [[1](#_ENREF_1)] |
| LN01/*ΔleuB* | A1160, *ΔleuB::pyr4* | This study |
| LN02/*leuB^C^* | A1160, *ΔleuB::pyr4*, *leuB*, *hph* | This study |
| LN03/*ΔleuB^AnleuB^* | A1160, *ΔleuB::pyr4*, *AnleuB*, *hph* | This study |
| LN04/*ΔAnleuB* | TN02A7, *ΔAnleuB::pyrG* | This study |
| LN05/*ΔhapX* | A1160, *ΔhapX::hph* | This study |
| LN06/*ΔsreA* | A1160, *ΔsreA::hph* | This study |
| LN07/*ΔleuBΔhapX* | A1160, *ΔleuB::pyr4*, *ΔhapX::hph* | This study |
| LN08/*ΔleuBΔsreA* | A1160, *ΔleuB::pyr4*, *ΔsreA::hph* | This study |
| LN09/LeuB^GFP^ | A1160, *leuB::gfp::pyrG* | This study |
| LN10/LeuB^GFP,RFP-NLS^ | A1160, *leuB::gfp::pyrG*, *rfp-nls*, *hph* | This study |
| LN11/WT^LeuB-FALG^ | A1160, *AngpdA(p)-leuB-flag*, *pyr4* | This study |
| LN12/*leuB^866^* | A1160, *ΔleuB::pyr4*, *leuB^866^*, *hapX-flag*, *hph* | This study |
| LN13/*leuB^815^* | A1160, *ΔleuB::pyr4*, *leuB^815^*, *hapX-flag*, *hph* | This study |
| LN14/*leuB^662^* | A1160, *ΔleuB::pyr4*, *leuB^662^*, *hapX-flag*, *hph* | This study |
| LN15/*leuB^C240A^* | A1160, *ΔleuB::pyr4*, *leuB^C240A^*, *hapX-flag*, *hph* | This study |
| LN16/*leuB^L717A^* | A1160, *ΔleuB::pyr4*, *leuB^L717A^*, *hapX-flag*, *hph* | This study |
| LN17/*leuB^P823A^*^,^ *^S833A^* | A1160, *ΔleuB::pyr4*, *leuB^P823A^*^,^ *^S833A^*, *hapX-flag*, *hph* | This study |
| LN18/*leuB^S846A^*^,^ *^D854A^* | A1160, *ΔleuB::pyr4*, *leuB^S846A^*^,^ *^D854A^* , *hapX-flag*, *hph* | This study |
| LN19/*ΔleuA* | A1160, *ΔleuA::pyr4* | This study |
| LN20/ WT^GFP-Atg8^ | A1160, *AngpdA(p)-* *gfp-atg8*, *hph* | This study |
| LN21/*ΔleuB*^GFP-Atg8^ | A1160, *AngpdA(p)-* *gfp-atg8*, *hph*，*ΔleuB::pyr4* | This study |

1. Jiang H, Shen Y, Liu W, Lu L (2014) Deletion of the putative stretch-activated ion channel Mid1 is hypervirulent in *Aspergillus fumigatus*. Fungal Genet Biol 62: 62-70.
