# Supplementary material for: The Zn2Cys6-type transcription factor LeuB cross-links regulation of leucine biosynthesis and iron acquisition in Aspergillus fumigatus
Source: PLoS Genet. 2018 Oct 26;14(10):e1007762. doi: 10.1371/journal.pgen.1007762 (PMC6221358; doi:10.1371/journal.pgen.1007762)
Supplement: S3 Table — (DOCX) [file pgen.1007762.s009.docx]

| **S3 Table. Primers used in this study.** | |
| --- | --- |
| **Primer name** | **Primer sequence 5' - 3'** |
| **For gene deletion** | |
| LeuB P1 | GTTCTACTTCAGCAGGTTTCGG |
| LeuB P2 | AATACCTGATCCGGTCATAGCA |
| LeuB P3 | CGATTAAGTTGGGTAACGCCATTTTGAAGCCTACCATAGCCAC |
| LeuB P4 | ATAAGTAGCCAGTTCCCGAAAGC AAACTATCGCCTGAAGTACCTGG |
| LeuB P5 | TGTAGGTCGCGTAGGTGTAGC |
| LeuB P6 | TCATCTCCTGCCTCCCTAATC |
| LeuB S1 | AGGAGTCGGAATGCTGAGATG |
| LeuB S2 | CCAGAACAGTAATGGTGATACGC |
| SreA P1 | AAAGAAAACATCAGGAACC |
| SreA P2 | ATAGCAACAGCACCAGACA |
| SreA P3 | GCCTGTGTGTAGAGATACAAGGGAATTCCGCCACTATCAACCCTCACC |
| SreA P4 | TAAGCGCCCACTCCACATCTCCACTCGATGCGCAGTCCCTTCACTATC |
| SreA P5 | CTAACATCACCTCCAGCAT |
| SreA P6 | ACTCCGACCTCAACCTCAT |
| SreA S1 | ACGTGTTGTGCCTGCCTTG |
| SreA S2 | GGTGTCGCTTTGTGGTGTT |
| HapX P1 | TCCAGGACTGATAACCACG |
| HapX P2 | AGTAAGTAGTTGCTGTGCG |
| HapX P3 | GCCTGTGTGTAGAGATACAAGGGAATTCGATTACGGATGATGAGACT |
| HapX P4 | TAAGCGCCCACTCCACATCTCCACTCGATTTATCGCATCTCTGCTTG |
| HapX P5 | GAAGTGATGGTTAGTGGTG |
| HapX P6 | CTTTTCTGGGGTCTGGTCT |
| HapX S1 | ACCAAAACCAGGCAGGAAA |
| HapX S2 | GGCAAATCGGGAAGTGAAA |
| AnleuB P1 | AAAGACTTCAAGCAGCAAACAGA |
| AnleuB P2 | GTCTCCCGCTTCACTCGTCTA |
| AnleuB P3 | GGTGAAGAGCATTGTTTGAGGCCACGGGGCTGGTGACGACAGAAGCA |
| AnleuB P4 | CATCAGTGCCTCCTCTCAGACAGTATCTGTTTATGTTTTCGCTGTCTT |
| AnleuB P5 | AACCTCTTATGCCCCATCAAC |
| AnleuB P6 | TCAAGTCTTACCATCCCAGTTCC |
| AnleuB S1 | CAGACCCCGAAACAAGAAAAC |
| AnleuB S2 | AGAGGTGCTGCTTGTAGGAAAA |
| LeuA P1 | ATTGCTCTGGCTTATGTGCG |
| LeuA P2 | TGACTGGGTTGCCTGACTGT |
| LeuA P3 | CGATTAAGTTGGGTAACGCCAGAGGGGCAAAGGCGAGCGCAAAG |
| LeuA P4 | ATAAGTAGCCAGTTCCCGAAAGCATGTCTCTATGCCGTGATTGTC |
| LeuA P5 | CAGGGATTGGGCTGTATGTT |
| LeuA P6 | TTCCTGAGCGCACAGTATGG |
| LeuA S1 | TGGCGTGACCAGTAAGGATGT |
| LeuA S2 | GAATCTTGGCGTTGCGGTAG |
| pyr4 F | TGGCGTTACCCAACTTAATCG |
| pyr4 R | GCTTTCGGGAACTGGCTACTTAT |
| De-pyr4 F | GCAGAACCTGAACGGCATTAC |
| De-pyr4 R | GGTCCAAGTGGAAGTAGGTAGTGAC |
| pyrG F | GCCTCAAACAATGCTCTTCACC |
| pyrG R | CTGTCTGAGAGGAGGCACTGATG |
| hph F | GAATTCCCTTGTATCTCTACACACAGGC |
| hph R | TCGAGTGGAGATGTGGAGTGGGCGCTTA |
| **For truncation** | |
| LeuB-trunc F | GCATTTTACGTGGAAGTTCTTGT |
| LeuB-866 R | TCACTAGCCCTGTGGGATCATGGTAGCG |
| LeuB-815 R | TCACTAATTGCGGCCTTTGGTCTGTGCG |
| LeuB-662 R | TCACTAAGGATCGTCAAAGAATGCCGAC |
| **For site directed mutagenesis** | |
| LeuB-C240A F | GCTCCGAGCTGATGTGATTCAAGACCCATGGACAGATTGTTCACG |
| LeuB-C240A R | CGTGAACAATCTGTCCATGGGTCTTGAATCACATCAGCTCGGAGC |
| LeuB-L717A F | CTGCACACTCCTCAAGGCCTGCAAGAGTTTC |
| LeuB-L717A R | GAAACTCTTGCAGGCCTTGAGGAGTGTGCAG |
| LeuB-P823A, 833A F | CCTCAAGAACGCGACAAATCCCGACTCGAACTCGGAATCTGCAGCGTCGTCAACC |
| LeuB-P823A, 833A R | GGTTGACGACGCTGCAGATTCCGAGTTCGAGTCGGGATTTGTCGCGTTCTTGAGG |
| LeuB-S846A, D854A F | GGCGCACCTCGGCCTCCACACCCGGCCTCCCGGCTGCCCCAAGTCTCGC |
| LeuB-S846A, D854A R | GCGAGACTTGGGGCAGCCGGGAGGCCGGGTGTGGAGGCCGAGGTGCGCC |
| **For gene label** | |
| LeuB-gfp P1 | ATCATCCCTTTCTTCCATTCCT |
| LeuB-gfp P2 | ATACCCATCTGCTCAACTCGC |
| LeuB-gfp P3 | CCAGCGCCTGCACCAGCTCCGGCGATAGTTTGTGGCTCCATCCCT |
| LeuB-gfp P4 | CATCAGTGCCTCCTCTCAGACAGTGAAGTACCTGGCCGCGGCGTAATC |
| LeuB-gfp P5 | TGCTCTGATAGGTTCCACCATG |
| LeuB-gfp P6 | GAGTCGGAAGAACCTCCACAA |
| gfp-pyrG F | GGAGCTGGTGCAGGCGCTGG |
| gfp-pyrG R | CTGTCTGAGAGGAGGCACTGATG |
| RFP F | CGGATATCATGGCCTCCTCCGAGGACGTC |
| RFP R | CATTCCAGTCGAGTAGCCGGCGCCGGTGGAGTGGCGG |
| NLS (AnStuA) F | GGCTACTCGACTGGAATG |
| NLS (AnStuA) R | CGGATATCTTAACGACGAGCACTTATCAGAC |
| LeuB-flag F | CCTTTAATCAAGCTTATCGATATGAATCATACCAACAACCACAG |
| LeuB-flag R | CATTCCCGGGGATCCCTCGAGGGCGATAGTTTGTGGCTCCATCC |
| Flag F | CTCGAGGGATCCCCGGGAATG |
| Flag R | CTCGAGGTCGACGGTATCGATGACATTCCTTTTACCCGGGCTA |
| GFP F | CCTTTAATCAAGCTTATCGATATGAGTAAAGGAGAAGAACTTTTCAC |
| GFP R | CTGGATCTCGGAGATTTTGTATAG |
| Atg8 F | CTATACAAAATCTCCGAGATCCAGCGGTCGAAGTTCAAGGACGAG |
| Atg8 R | CTCGAGGTCGACGGTATCGATTCAGCAGTCACCGAAAGTGTTCTC |
| **For protein expression** | |
| Ex-LeuB P1 | GGAATTCCATATGCACCATCATCATCATCATATCGTCAACGTTCCCAGCT |
| Ex-LeuB P2 | GAATCACATCACATCGGAGCTTTTGTTGTCGACATTCG |
| Ex-LeuB P3 | CTCCGATGTGATGTGATTC |
| Ex-LeuB P4 | GGAATTCGATGGCGGACGGTGTGTGAT |
| **For EMSA** | |
| pEMSA | Cy5-AGCACGTGGTCGAAAG |
| EMSA-hapX F | AGCACGTGGTCGAAAGGTGGGAGGGTTGTAATAG |
| EMSA-hapX R | AGCACGTGGTCGAAAGAGGAAAAGACGGCGAATA |
| EMSA-gdhA F | AGCACGTGGTCGAAAGCACGAAAGATATGGATGG |
| EMSA-gdhA R | AGCACGTGGTCGAAAGTCGATACTTTAAGGTCAC |
| EMSA-MuhapX F | ACCGCTGAAATCAGGAAG |
| EMSA-MuhapX R | CTTCCTGATTTCAGCGGT |
| EMSA-leuA F | AGCACGTGGTCGAAAGCAAGTCAATCAGAGCACCTGC |
| EMSA-leuA R | AGCACGTGGTCGAAAGTCGGTCCGGTCAGCAGAGATT |
| EMSA-leu2A F | AGCACGTGGTCGAAAGGTATGAGTGTTTTGTATAGCTCC |
| EMSA-leu2A R | AGCACGTGGTCGAAAGCTGAAGGACAATTGCAGACGAGTG |
| EMSA-bat2 F | AGCACGTGGTCGAAAGCGTGACAGTTAGTTCCAAAAGCTG |
| EMSA-bat2 R | AGCACGTGGTCGAAAGCTCAATCAGAGTGTTTCTTTGTTG |
| EMSA-lysF F | AGCACGTGGTCGAAAGCGATCAAGATGGACGAACCATTG |
| EMSA-lysF R | AGCACGTGGTCGAAAGGAAATGGCCCAAGACAAGTAGATG |
| EMSA-areA F | AGCACGTGGTCGAAAGGTCTCTGTTTAGCTTGACAGCTTG |
| EMSA-areA R | AGCACGTGGTCGAAAGCAGAATAACAAGATTCGCCATTC |
| EMSA-metR F | AGCACGTGGTCGAAAGCCACTATTCGCATACCTCATCGTG |
| EMSA-metR R | AGCACGTGGTCGAAAGCTGTGCCTCATTGACTTTTCCG |
| EMSA-mrsA F | AGCACGTGGTCGAAAGCCTGTAGTGCTAGCATATCTAGG |
| EMSA-mrsA R | AGCACGTGGTCGAAAGCTAACATGATATACTACTGTCTC |
| EMSA-estB F | AGCACGTGGTCGAAAGCGTAAGATCATTGTGACCGCAG |
| EMSA-estB R | AGCACGTGGTCGAAAGGTGGGAATGGCCAGTCAAAACC |
| EMSA-sidJ F | AGCACGTGGTCGAAAGCCAAGTGCAGCAGCACATGAGG |
| EMSA-sidJ R | AGCACGTGGTCGAAAGCGTTTTGATTGTGGTTGAAGCTGG |
| **For complementation** | |
| ComLeuB F | GCATTTTACGTGGAAGTTCTTGT |
| ComLeuB R | TGTGCCTATCCTTGTTGTTGTTT |
| ComAnleuB F | CTCCCGCTTCACTCGTCT |
| ComAnleuB R | TAGCCCTAATAAGCATCAAAC |
| hph-Spe I F | CGGACTAGTGAATTCCCTTGTATCTCTAC |
| hph-Spe I R | CGGACTAGTTCGAGTGGAGATGTGGA |
| **For RT-PCR** | |
| RT-Tub A F | ACGTTACCTCACCTGCTCTGC |
| RT-Tub A R | GATGTTGTTGGGAATCCACTCA |
| RT-LeuB-trunc F | CTCTGTACGACTGGACGCTATC |
| RT-LeuB-866 R | GCCCTGTGGGATCATGGTAGCG |
| RT-LeuB-815 R | ATTGCGGCCTTTGGTCTGTGCG |
| RT-LeuB-662 R | AGGATCGTCAAAGAATGCCGAC |
| RT-LeuB 920 R | GGCGATAGTTTGTGGCTCCAT |
| **For Northern blot** | |
| LeuB F | CTGGACTATCATCAGCGTTG |
| LeuB R | GTCAAACAAGAGCGACATGC |
| LeuA F | GGTCTCACATACTTCGGC |
| LeuA R | TTCACGCACATCGGCAAG |
| Leu2A F | GACGAAGCTCTTAACGCC |
| Leu2A R | CTCGATCACATTGCGGAC |
| Ilv5 F | TCTTGCAAAGCATCAAATCC |
| Ilv5 R | GAAGGTGGTCTCGTAGAG |
| GdhA F | GATCAACCGTGGTTACCG |
| GdhA R | ACCACCAGTCACCCTGC |
| HapX F | TCGGTGGAAAGAAGTGCC |
| HapX R | CGACGATGTATTGTTATTGG |
| SidA F | AACTACCTCCACCAGAAG |
| SidA R | GAACGGCAATGTTGTAAG |
| MirB F | AAGCCGAGAAAAAGGGGG |
| MirB R | AACCCAGATGAAGCCCAG |
